# Cardiac glycoside ouabain efficiently targets leukemic stem cell apoptotic machinery independent of cell differentiation status

**DOI:** 10.1186/s12964-023-01317-8

**Published:** 2023-10-12

**Authors:** Jirarat Poohadsuan, George A. O’Doherty, Weerapat Owattanapanich, Smith Kungwankiattichai, Yon Rojanasakul, Surapol Issaragrisil, Sudjit Luanpitpong

**Affiliations:** 1 Siriraj Center of Excellence for Stem Cell Research, Faculty of Medicine Siriraj Hospital, Mahidol University, 2 Siriraj Hospital, Bangkoknoi, Bangkok, 10700 Thailand; 2 Department of Chemistry and Chemical Biology, Northeastern University, Boston, MA USA; 3 Division of Hematology, Department of Medicine, Faculty of Medicine Siriraj Hospital, Mahidol University, Bangkok, Thailand; 4 Center of Excellence of Siriraj Adult Acute Myeloid/Lymphoblastic Leukemia, Faculty of Medicine Siriraj Hospital, Mahidol University, Bangkok, Thailand; 5 Department of Pharmaceutical Sciences, West Virginia University, Morgantown, WV USA; 6 WVU Cancer Institute, West Virginia University, Morgantown, WV USA

**Keywords:** Cardiac glycosides, Ouabain, Acute myeloid leukemia, Leukemic stem cell, Apoptosis, Cell cycle, Mcl-1, c-Myc

## Abstract

**Background:**

Acute myeloid leukemia (AML) is an aggressive hematologic malignancy characterized by an accumulation of immature leukemic myeloblasts initiating from leukemic stem cells (LSCs)—the subpopulation that is also considered the root cause of chemotherapy resistance. Repurposing cardiac glycosides to treat cancers has gained increasing attention and supporting evidence, but how cardiac glycosides effectively target LSCs, e.g., whether it involves cell differentiation, remains largely unexplored.

**Methods:**

Digoxin, a user-designed digitoxigenin-α-L-rhamnoside (D6-MA), and ouabain were tested against various human AML-derived cells with different maturation phenotypes. Herein, we established two study models to specifically determine the effects of cardiac glycosides on LSC death and differentiation—one allowed change in dynamics of LSCs and leukemic progenitor cells (LPCs), while another maintained their undifferentiated status. Regulatory mechanisms underlying cardiac glycoside-induced cytotoxicity were investigated and linked to cell cycle distribution and apoptotic machinery.

**Results:**

Primitive AML cells containing CD34^+^ LSCs/LPCs were very responsive to nanomolar concentrations of cardiac glycosides, with ouabain showing the greatest efficiency. Ouabain preferentially induces caspase-dependent apoptosis in LSCs, independent of its cell differentiation status, as evidenced by (i) the tremendous induction of apoptosis by ouabain in AML cells that acquired less than 15% differentiation and (ii) the higher rate of apoptosis in enriched LSCs than in LPCs. We sorted LSCs and LPCs according to their cell cycle distribution into G0/G1, S, and G2/M cells and revealed that G0/G1 cells in LSCs, which was its major subpopulation, were the top ouabain responders, indicating that the difference in ouabain sensitivity between LSCs and LPCs involved both distinct cell cycle distribution and intrinsic apoptosis regulatory mechanisms. Further, Mcl-1 and c-Myc, which were differentially expressed in LSCs and LPCs, were found to be the key apoptosis mediators that determined ouabain sensitivity in AML cells. Ouabain induces a more rapid loss of Mcl-1 and c-Myc in LSCs than in LPCs via the mechanisms that in part involve an inhibition of Mcl-1 protein synthesis and an induction of c-Myc degradation.

**Conclusions:**

Our data provide new insight for repurposing cardiac glycosides for the treatment of relapsed/refractory AML through targeting LSCs via distinct cell cycle and apoptosis machinery.

Video Abstract

**Supplementary Information:**

The online version contains supplementary material available at 10.1186/s12964-023-01317-8.

## Background

Acute myeloid leukemia (AML), the most common acute leukemia in adults, is an aggressive hematologic malignancy characterized by an accumulation of immature leukemic myeloblasts arising from differentiation blockage. Despite major advances in understanding its genetics and epigenetics, the standard of care for AML using cytarabine and anthracyclines as backbone chemotherapy has remained unchanged for more than four decades. The treatment outcome of AML is generally poor, with a 5-year survival rate of approximately 30% overall and less than 5% among patients aged 65 years and older [1, 2]. Relapse is a common scenario in AML, occurring in 40 − 50% of young adults and in the great majority of elderly patients even after complete remission, and is associated with the dismal outcomes [3]. Leukemic stem cells (LSCs), which are capable of self-renewal and continuously generating more mature progenies, such as leukemic progenitor cells (LPCs) and leukemic blasts (LBs), are resistant to standard therapies and are the root of relapse in AML [4, 5]. Accordingly, there is an urgent and unmet need for novel therapies, particularly those targeting LSCs, to improve clinical outcome and enable long-term control of disease.

Drug repurposing, also called drug repositioning, is a strategy for identifying new indications for approved or investigational drugs other than its original intended purpose. This strategy significantly shortens the long-term process of drug development, as in many cases the safety assessment in preclinical models and humans have been completed [6]. Cardiac glycosides are a diverse family of naturally derived compounds approved for the treatment of congestive heart failure and certain irregular heartbeats, primarily as Na^+^/K^+^ ATPase pump inhibitors [7]. In addition to epidemiological studies describing reduced breast cancer and leukemia diagnoses and breast cancer reoccurrence in heart failure patients on cardiac glycoside therapy [8, 9], several in vitro studies have shown the cytotoxicity of cardiac glycosides against multiple human solid and hematologic tumor cell lines [10–13], thus supporting their potential application as anticancer drugs. In a recent high-throughput drug screening in AML 8227 cells, cardiac glycosides strophanthidin, ouabain, and digoxin in the micromolar range for 6 days were shown to be effective against all leukemic populations tested, including CD34^+^CD38^−^ LSCs, CD34^+^CD38^+^ LPCs, and CD15^+^ mature myeloid cells [14]. However, at its lower concentrations in the nanomolar range, the screened cardiac glycosides were shown to have relatively high toxicity towards LSCs, suggesting that cardiac glycosides are good candidates for targeting primitive AML cells. Thus far, the mechanisms underlying the anti-leukemic effects of cardiac glycosides, particularly those that address their cytotoxic effects against LSCs, which are important for the future rational therapeutics, remain to be elucidated.

Digoxin, digitoxin, and ouabain are among the best described anticancer cardiac glycosides [15, 16]. Various synthetic analogs of digitoxin with improved cytotoxicity and selectivity toward tumor cells have been developed by manipulating the chemical structure of its saccharide moiety [17, 18]. In this regard, we previously demonstrated that digitoxigenin-α-L-rhamnoside (D6-MA), a user-designed L-sugar monosaccharide analog of digitoxin, exhibited greater potency than digitoxin itself in eliminating non-small cell lung cancer (NSCLC) cells under attached and detached (undergoing anoikis) conditions [19–21]. In the present study, we first evaluated the effects of digoxin, digitoxin analog D6-MA, and ouabain in various subtypes of human AML cells containing different proportions of LSCs, LPCs, and LBs in comparison to peripheral blood mononuclear cells (PBMCs) and CD34^+^ hematopoietic stem/progenitor cells (HSPCs) obtained from healthy donors. Importantly, we established two study models to specifically determine the effects of cardiac glycosides on LSC death and differentiation, as overcoming differentiation blockage has been a promising strategy for AML therapy [22, 23]. The underlying mechanisms of action were further investigated using the most potent cardiac glycoside tested, which in this case was ouabain. Our findings could lay a basis for the repurposing of cardiac glycosides for AML therapy and lend insight into whether and how cardiac glycosides affect LSCs.

## Methods

### Subjects and ethics statement

This study was approved by the Siriraj Review Board (COA Nos. Si 101/2015 and Si 564/2018) and was in accordance with the Helsinki Declaration of 1975. Human peripheral blood was collected from healthy donors and AML patients after informed consent. Umbilical cord blood was collected from a healthy newborn at birth after informed consent from the mother.

### Reagents

**Fig. 1:**
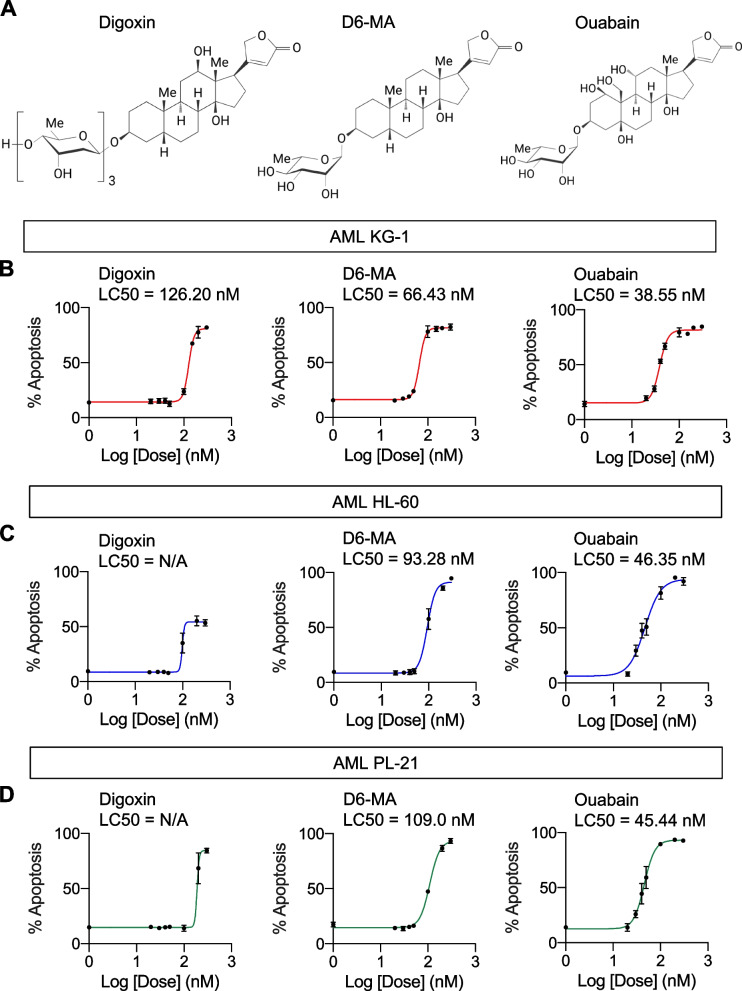
Cardiac glycosides induce apoptosis in a panel of human AML-derived cells. **A** Structures of digoxin, user-designed digitoxin analog D6-MA, and ouabain. **B** − **D** Dose–response curves generated from the percentage of apoptosis as evaluated by Annexin V/7-AAD assay after cardiac glycoside treatment (0 − 300 nM) for 48 h in various human AML-derived cells, including KG-1 (M0) (**B**), HL-60 (M2) (**C**), and PL-21 (M3) (**D**) cells. LC50 were then calculated from the plots and compared among different AML cells and treatments. Data are mean ± SD (*n* = 3)

Digoxin and ouabain were obtained from Sigma-Aldrich (St. Louis, MO). D6-MA was synthesized using a de novo Pd-catalyzed glycosylation method for unique carbohydrate synthesis as previously described [19, 21]. The chemical structures of tested cardiac glycosides were shown in Fig. 1A. Caspase inhibitors zVAD-IETD and zVAD-LEHD were obtained from Cell Signaling Technology (Danvers, MA), while Mcl-1 inhibitor II was from Calbiochem (Sigma-Aldrich). Primary and secondary antibodies were obtained from Cell Signaling Technology (Danvers, MA), unless otherwise specified.

### Cell culture and patient-derived primary cells

Human AML cell lines, KG-1, HL-60, PL-21, and Kasumi-3 cells, were obtained from Japanese Collection of Research Bioresources (JCRB) Cell Bank (Osaka, Japan) and cultured in RPMI 1640 medium supplemented with 10% fetal bovine serum (FBS), 100 U/mL penicillin, and 100 µg/mL streptomycin at 37 °C, 5% CO_2_ and 95% relative humidity. Mycoplasma contamination was routinely tested by using MycoAlert™ PLUS Mycoplasma Detection Kit (Lonza, Cologne, Germany), and any cell lines found positive were discarded. Patient-derived primary AML cells were obtained from PBMCs of relapsed patients, which were enriched over a Lymphoprep gradient (STEMCELL Technologies, Vancouver, Canada) (see Additional file 1: Table S1 for clinical characterization). The primary AML cells were then cultured in StemSpan medium (STEMCELL Technologies) supplemented with 10 ng/mL interleukin (IL)-3, IL-6 and granulocyte colony-stimulating factor (G-CSF), 25 ng/mL thrombopoietin (TPO), 50 ng/mL stem cell factor (SCF) and FLT3 ligand, which promoted the growth and expansion of LSCs (Additional file 1: Table S2). PBMCs from healthy donors were used as normal blood cells.

### Purification of normal CD34^+^ HSPCs

Mononuclear cells were enriched from human cord blood and G-CSF-mobilized peripheral blood over a Lymphoprep gradient and CD34^+^ HSPCs were subsequently isolated using CD34 MicroBead Kit and MS Column (Miltenyi Biotec, Bergisch Gladbach, Germany) according to the manufacturer protocol. Notably, the blood mobilization process was performed by daily injection of 10 μg/kg/day G-CSF (filgrastim; Neuprogen, Amgen Inc, Thousand Oaks, CA, USA) for 4 days with apheresis on the following day. The purity of enriched CD34^+^ was > 90% as evaluated by flow cytometry (Additional file 1: Fig. S1).

### Flow cytometric analysis of AML subpopulations

AML cells were stained with APC-conjugated CD34 and PerCP-conjugated CD38 antibodies (BD Biosciences, San Jose, CA) for 15 min at room temperature and analyzed using FACSCanto (BD Biosciences). Live cells were characterized into LSCs (CD34^+^CD38^−^), LPCs (CD34^+^CD38^+^), pre-LBs (CD34^−^CD38^+^), and LBs (CD34^−^CD38^−^), as previously described [5, 24]. Cells were also stained using BV605-conjugated CD15 antibody to identify the terminally differentiated CD15^+^ blasts [14]. TIM3, which is highly expressed in AML LSCs, but not normal HSPCs [25–27], was additionally used to verify LSCs in the tested AML cell lines and primary AML cells (Additional file 1: Fig. S2).

### Apoptosis assay

Apoptosis was evaluated by Annexin V/7-AAD assay. Cells were washed and stained with PE-conjugated Annexin V and 7-AAD in binding buffer supplemented with 5 mmol/L calcium chloride for 15 min at room temperature. Samples were immediately analyzed by the FACSCanto flow cytometer (BD Biosciences) to identify the percentage of apoptosis, which was calculated from the combination of Annexin V^+^/7-AAD^−^ (early apoptosis) and Annexin V^+^/7-AAD^+^ (late apoptosis) fractions. Annexin V^−^/7-AAD^+^ cells were considered necrosis. Hoechst 33342 assay was used to validate the apoptosis by Annexin V/7-AAD assay. Cells were incubated with 10 μg/mL Hoechst 33342 for 30 min and analyzed for apoptosis by scoring of cells having condensed (brighter than non-apoptotic) and/or fragmented nuclei by fluorescence microscopy (Eclipse Ti-U with NiS-Elements, Nikon, Tokyo, Japan). The apoptotic index was then calculated as the percentage of cells with apoptotic nuclei over the total number of cells.

### Establishment of LSC study models

We used KG-1 cells, which naturally contained approximately 20% LSCs, to further establish two study models to determine the effect of cardiac glycosides on LSC death and differentiation. In model 1, LSCs were enriched by fluorescence activated cell sorting (FACS) using FACSAria (BD Biosciences) and cultured in regular complete RPMI 1640 medium to allow the differentiation of LSCs into LPCs. At three days after sorting where the ratio of LSC:LPC reached 1:1, cells were treated with ouabain and its effect on cell death, as defined by Annexin V^+^ cells, and the proportion of LSCs, LPCs, and CD15^+^ blasts in live (Annexin V^−^) cells were evaluated. In model 2, we performed FACS to obtain LSC and LPC fractions with the > 90% purity. Then, enriched LSCs were cultured in StemSpan medium (STEMCELL Technologies), which specifically maintained and promoted the expansion of LSCs, while enriched LPCs were cultured in regular RPMI 1840 medium, and the effects of ouabain against cell death in LSCs and LPCs were evaluated based on Annexin V^+^ cells. Notably, 7-AAD was not used in these experiments due to the spectral overlap with PerCP.

### Clonogenic assay

Base methylcellulose (MC) medium (MethoCult™ H4100; STEMCELL Technologies) was used to enrich the cancer stem cell subpopulation as previously described [28]. Briefly, a total of 2000 − 5000 cells were cultured in 1% MC in 6-well plates for 14 days and colonies were scored under an inverted microscope (Eclipse Ti-U).

### Cell cycle analysis

Cells were plated in 6-well plates at a density of 1 × 10^6^ cells per mL and starved in serum-free medium overnight. Cells were then incubated in the complete RPMI 1640 medium for 24 h and cell cycle analysis was performed using propidium iodide (PI) DNA dye. Briefly, cells were fixed in cold 70% ethanol for 1 h, washed twice, and stained with PI (10 µg/mL) for 10 min, after which at least 10,000 cells were analyzed by BD FACSCanto. Cell cycle distribution in sub-G1, G0/G1, S, and G2/M phases was analyzed by using FlowJo (v10.4.1) software. For staining of viable cells for cell cycle sorting, Hoechst 33342 DNA dye (0.5 µg/mL), which allowed the staining of unfixed samples, was used instead of PI.

### CRISPR/Cas9-mediated *MCL1* knockdown

Lentiviral plasmids carrying inducible single guided RNA (sgRNA) sequence against human *MCL1* and Cas9 were kind gifts of Prof. Marco Herold (Addgene #85530) [29] and Prof. Feng Zhang (Addgene #52962) [30]. Lentiviral production was performed using HEK293T packaging cells (American Type Culture Collection, ATCC; Manassas, VA) in conjunction with pCMV.dR8.2 dvpr lentiviral packaging and pCMV-VSV-G envelope plasmids (Addgene #8454 and 8455) [31]. Cells were first transfected with viral particles containing sgRNA with fluorescent GFP reporter in the presence of hexadimethrine bromide (HBr; 8 μg/mL) for 48 h and sorted for GFP-positive cells by FACSAria, hereafter designated as iMcl-1 cells. iMcl-1 cells were then transfected with Cas9 viral particles and selected with blasticidin (2 μg/mL) for 7 days. iMcl-1/Cas9 cells were then treated with doxycycline (DOX; 1 μg/mL) for 3 days for an induction of sgRNA or left untreated (control) and analyzed for Mcl-1 by Western blotting prior to use.

### shRNA-mediated *MYC* knockdown

Retroviral transduction particles carrying short hairpin RNA (shRNA) sequence against human *MYC* (shMYC; Addgene #15662), which was a kind gift of Prof. Martin Eilers [32], were used to inhibit c-Myc expression in AML cells. In brief, cells were incubated with retroviral particles in the presence of HBr for 36 h and cultured in puromycin-containing medium (1 μg/mL) for 28 days to generate the stable shMYC cells. c-Myc level was evaluated by Western blotting prior to use.

### Western blotting

Cells were incubated in lysis buffer (Cell Signaling Technology) containing protease inhibitor mixtures (Roche Molecular Biochemicals, Indianapolis, IN) and phenylmethylsulfonyl fluoride (PMSF) at 4 °C for 30 min. The protein concentration was determined using bicinchoninic acid (BCA) assay (Pierce Biotechnology, Rockford, IL, USA). Proteins (30 − 60 µg) were separated by 12% SDS-PAGE and transferred onto PVDF membranes. Membranes were blocked with 5% non-fat milk in TBST for 1 h at room temperature and incubated with appropriate primary antibodies overnight at 4 °C, followed by secondary antibodies for 1 h at room temperature. Immunoblots were detected by Immunobilon Western Chemiluminescent HRP substrate (Millipore, Burlington, MA) and visualized on an ImageQuant^TM^ digital imager (GE Healthcare, Pittsburgh, PA).

### RNA isolation and quantitative PCR

Total RNA was prepared using TRIzol reagent (Invitrogen, Carlsbad, CA, USA) according to the manufacturer’s protocol, and cDNA was prepared using SuperScript III First-Strand Synthesis System and oligo (dT) primers (Invitrogen). qPCR analysis was carried out on a CFX384 Real-Time PCR (BioRad, Hercules, CA, USA) using a SYBR Green PCR Master Mix (Applied Biosystems, Waltham, MA). Initial enzyme activation was performed at 95 °C for 10 min, followed by 40 cycles of denaturation at 95 °C for 15 s and primer annealing/extension at 60 °C for 1 min. Relative expression of each gene was normalized against the housekeeping *GAPDH* gene product.

### Cycloheximide-chase assay

Cells were treated with cycloheximide (CHX; 10 μg/mL) to inhibit new protein synthesis for various times (0–16 h) to follow the degradation of protein by Western blot analysis. Protein level was evaluated at various times, and protein half-life (t_1/2_) was calculated from the plot of natural log (Ln) of protein level versus time using linear regression by Prism 8 software by the following equation: t_1/2_ = [Ln(0.5)]/slope (GraphPad Prism, Boston, MA) [33].

### Statistical analysis

Data represent mean ± SD from at least three independent experiments. Statistical analysis was performed by two-sided, unpaired Student's *t*-test or one-way ANOVA followed by Tukey’s multiple comparison test at a significance level of *P* < 0.05 (GraphPad Prism).

## Results

### Cytotoxicity of cardiac glycosides in various human AML cells

**Fig. 2:**
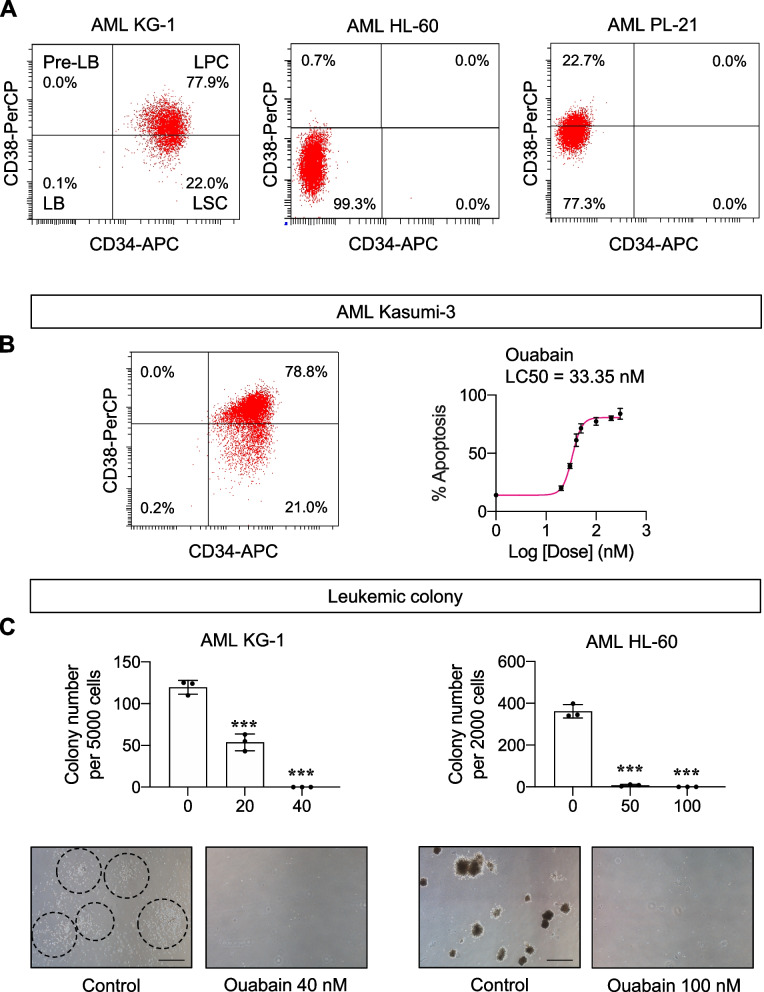
Ouabain preferentially targets primitive AML cells. **A** Profiles of different AML subpopulations, including CD34^+^CD38^−^ LSCs, CD34^+^CD38^+^ LPCs, CD34^−^CD38^+^ pre-LBs, and CD34^−^CD38^−^ LBs in human AML-derived KG-1, HL-60, and PL-21 cells, by flow cytometry. **B** Human AML-derived Kasumi-3 (M0) cells exhibited a similar profile of AML subpopulations as KG-1 cells (left) and dose–response curves upon ouabain treatment (0 − 300 nM) for 48 h (right). **C** Clonogenic assay in KG-1 (left) and HL-60 (right) with or without ouabain at the indicated concentrations after 14 days of culture. (upper) Number of leukemic colonies was plotted. Data are mean ± SD (*n* = 3). ^***^P < 0.001 versus nontreated control; two-sided Student’s *t* test. (lower) Representative micrographs are shown. Scale bar = 500 μm

A panel of human AML cell lines with different phenotypes and FAB subtypes, including KG-1 (early myeloblasts; M0), HL-60 (promyelocytes; M2), and PL-21 (promyelocytes; M3) cells [34, 35], were treated with various concentrations (0 − 300 nM) of digoxin, D6-MA, and ouabain, and apoptosis was analyzed by Annexin V/7-AAD assay at 48 h. Additional file 1: Fig. S3 shows that apoptosis was the major mode of cell death induced by cardiac glycosides. Lethal concentration 50 (LC50), which caused half of the cells to undergo apoptosis, was then calculated from dose–response curves using the best-fit values from simple logistic regression. Figure 1B − D shows that the sensitivity of different AML cells to cardiac glycosides varied, with KG-1 cells being the most responsive cells tested. For example, while the LC50 of digoxin in HL-60 and PL-21 cells could not be estimated, its LC50 in KG-1 cells was at the nanomolar level (126.20 nM). The majority of KG-1 cells were CD34^+^, which were relatively less mature than CD34^−^ HL-60 and PL-21 cells (Fig. 2A), suggesting that cardiac glycosides preferentially target primitive AML cells. Ouabain was found to be the most potent agent herein—the LC50 of ouabain in KG-1 cells was 38.55 nM versus 126.20 nM for digoxin (*P* < 0.0001) and 66.43 nM for D6-MA (*P* < 0.0001). Notably, the higher sensitivity of AML cells to ouabain was more pronounced in HL-60 and PL-21 cells, likely due to their relative resistance to digoxin and D6-MA.

To further validate that primitive AML cells were very responsive to ouabain, CD34^+^ Kasumi-3 cells (early myeloblasts; M0) were treated with ouabain (0 − 300 nM), and apoptosis and subsequent LC50 were similarly evaluated at 48 h. The results showed that the LC50 of ouabain in Kasumi-3 cells was relatively low at the reported value of 33.35 nM (Fig. 2B), similar to KG-1 cells. Next, we evaluated whether ouabain could also influence colony-forming cells termed CFU-leukemic, the functional progenitors that support the self-renewal of AML blasts. Figure 2C shows that ouabain was capable of significantly inducing the loss of clonogenic potential of AML cells, both at the early and late myeloblast characteristics, thus confirming the antileukemic potential of ouabain, particularly against the self-renewing, primitive AML cells.

### Ouabain preferentially induces LSC cell death independent of the cell differentiation status

**Fig. 3:**
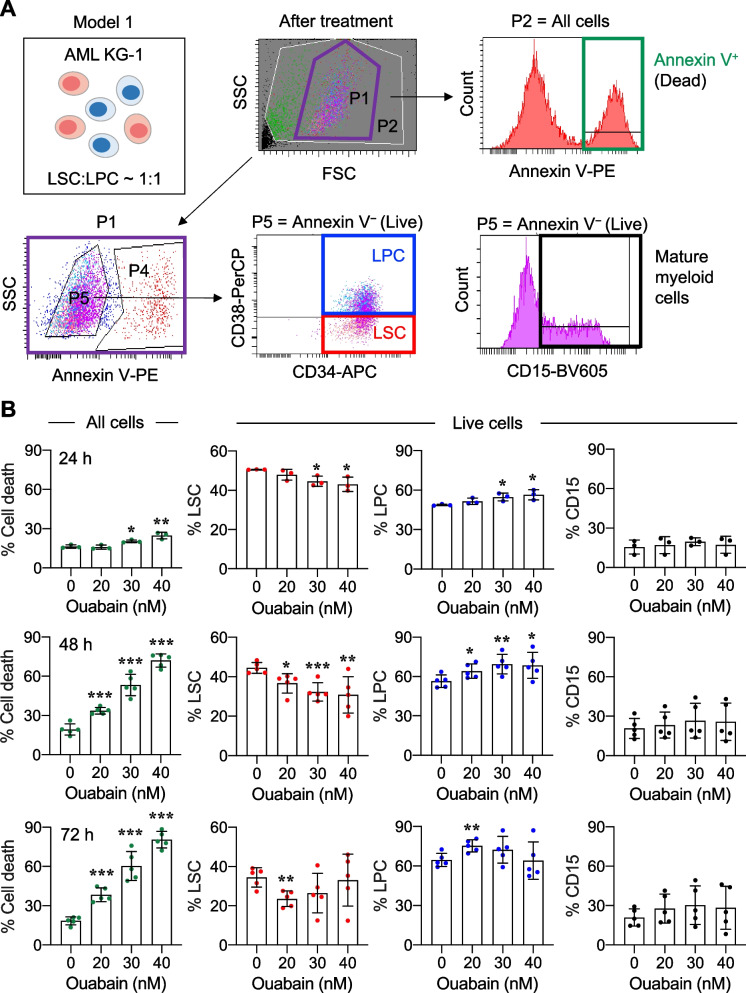
Ouabain induces the differentiation of LSCs into LPCs in viable AML cells. **A** Schematic illustration describing the established study model 1, which allowed the dynamic changes of LSCs and LPCs, along with the flow cytometry gating strategy to identify cell death in pooled AML cells and LSC/LPC differentiation in viable cells. **B** Pooled AML KG-1 cells containing approximately 50% LSCs and LPCs each were treated with ouabain (0 − 40 nM) for 24 − 72 h. (left) Percentage of cell death (Annexin V^+^) in pooled AML cells. (right) Percentage of CD34^+^CD38^−^ LSCs, CD34^+^CD38^+^ LPCs, and CD15^+^ blasts in live (Annexin V^−^) cells. Data are mean ± SD (*n* = 5). ^*^P < 0.05, ^**^P < 0.01, ^***^P < 0.001 versus nontreated control; two-sided Student’s *t* test

Primitive AML cells with sustained self-renewal generally include the early stem/progenitors CD34^+^CD38^−^ LSCs and CD34^+^CD38^+^ LPCs. Having demonstrated the potential of ouabain in targeting primitive AML cells, we next established two study models to specifically determine the effects of ouabain on LSC and LPC death and differentiation, as previous studies reported that the differentiation of AML cells by certain stimuli could induce apoptosis [36, 37]. In study model 1, LSCs and LPCs from AML KG-1 cells, that reached a ratio of 1:1 as described in methods section, were cultured in regular AML medium to allow the change in dynamics of LSCs and LPCs. After treatment with ouabain (0 − 40 nM) for various times (0 − 72 h), cell death was evaluated in the pooled population using Annexin V assay, as schematically shown in Fig. 3A. Then, the proportion of LSCs, LPCs, and the terminally differentiated CD15^+^ blasts was evaluated in the live (Annexin V^−^) cell fraction. Figure 3B shows that ouabain remarkably induced pooled AML cell death (Annexin V^+^) in a dose- and time-dependent manner, thus validating its cytotoxic effect against AML cells at an early stage. In viable AML cells, a subtle, yet significant, decrease in the LSC subpopulation was observed in parallel with a subtle increase in LPCs, while the percentage of CD15^+^ cells was insignificantly changed. The maximum induction of LSC differentiation was approximately 15% by 40 nM ouabain at 48 h, where its rate of apoptotic induction was 50%, suggesting that the cytotoxic effects of ouabain may not be directly related to cell differentiation.

**Fig. 4:**
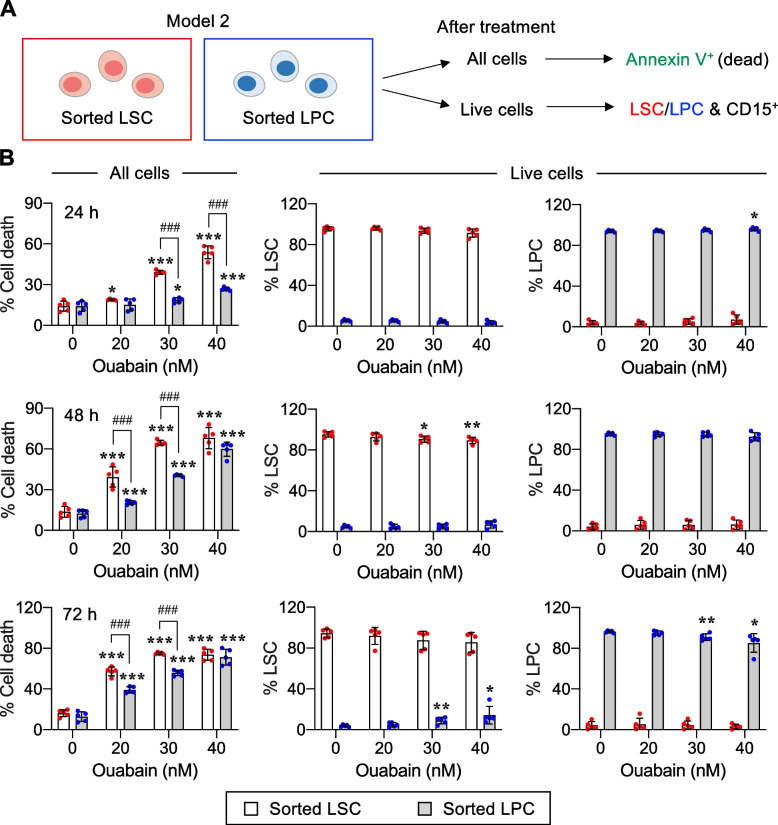
Ouabain exhibits greater cytotoxicity in LSCs than LPCs. **A** Schematic illustration describing the established study model 2, in which enriched LSCs and LPCs were separately cultured to maintain their undifferentiated status, along with the flow cytometry gating strategy to identify cell death in LSCs and LPCs. **B** Enriched LSCs and LPCs from AML KG-1 cells were similarly treated with ouabain (0 − 40 nM) for 24 − 72 h. (left) Percentage of cell death (Annexin V^+^) in LSCs or LPCs. (right) Percentage of CD34^+^CD38^−^ LSCs and CD34^+^CD38^+^ LPCs in live (Annexin V^−^) cells to confirm their undifferentiated status. Data are mean ± SD (*n* = 5). ^*^P < 0.05, ^**^P < 0.01, ^***^P < 0.001 versus nontreated control; ^###^*P* < 0.001 versus enriched LSCs; two-sided Student’s *t* test

To further rule out the involvement of cell differentiation in ouabain cytotoxicity, we established study model 2, in which enriched LSCs and LPCs were separately cultured in specific media to maintain their undifferentiated status (Fig. 4A). Enriched LSCs and LPCs were separately treated with ouabain (0 − 40 nM) for various times (0 − 72 h), and cell death and differentiation were similarly evaluated as in study model 1. Figure 4B shows that ouabain induced cell death in both LSCs and LPCs in a dose- and time-dependent manner. Remarkably, LSCs were more sensitive to ouabain than LPCs at all time points tested. The percentages of LSCs, LPCs, and CD15^+^ cells were confirmed to be unchanged in this model (see also Additional file 1: Fig. S4), thus validating the suitability of our test conditions and the dispensability of cell differentiation in ouabain cytotoxicity. Altogether, our results indicate that ouabain preferentially induces cell death in LSCs when compared to LPCs, independent of the cell differentiation status.

**Fig. 5:**
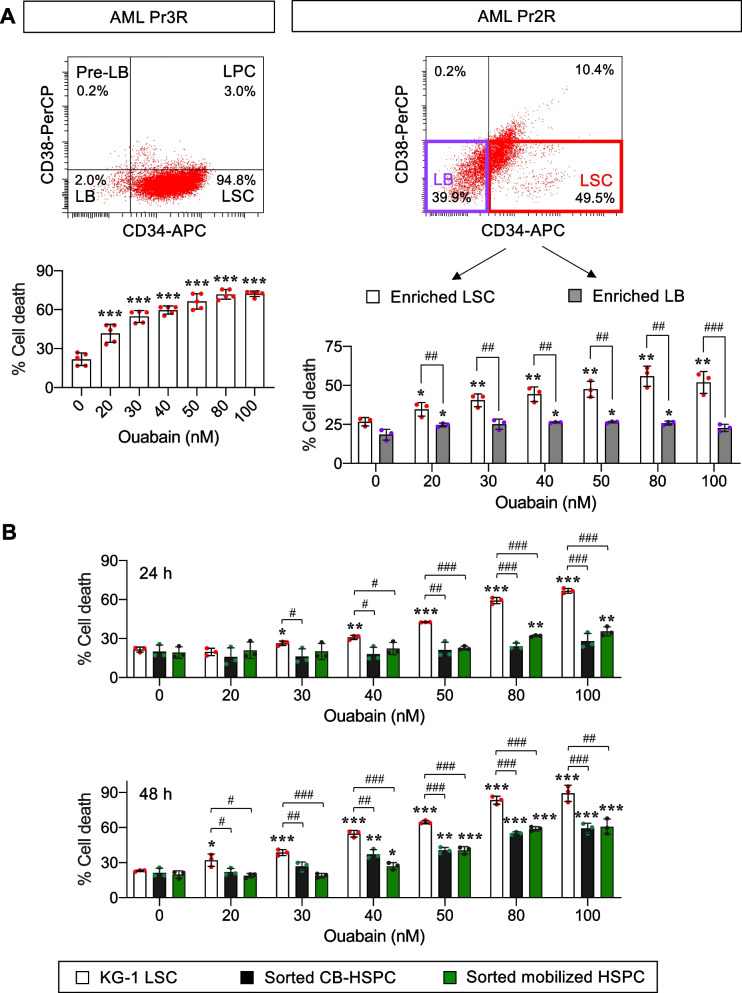
Ouabain exhibits greater cytotoxicity in LSCs than normal HSPCs. **A** Primary AML cells from relapsed patients, including Pr3R (left) and Pr2R (right) cells, were similarly treated with ouabain (0 − 100 nM) for 48 h. (upper) Profiles of different AML subpopulations, including CD34^+^CD38^−^ LSCs, CD34^+^CD38^+^ LPCs, CD34^−^CD38^+^ pre-LBs, and CD34^−^CD38^−^ LBs, in primary AML cells in StemSpan culture by flow cytometry. (lower) Percentage of cell death (Annexin V^+^) in pooled Pr3R AML cells or enriched Pr2R LSCs and LBs. Data are mean ± SD (*n* = 3 or 5). ^*^P < 0.05, ^**^P < 0.01, ^***^P < 0.001 versus nontreated control; ^*##*^*P* < 0.01, ^*###*^*P* < 0.001 versus ouabain-treated LSCs at the same concentration; two-sided Student’s *t* test. **B** Enriched LSCs from AML KG-1 cells and enriched normal HSPCs from cord blood (CB) and G-CSF-mobilized peripheral blood were similarly treated with ouabain (0 − 100 nM) for 24 − 48 h. Cell death was evaluated by Annexin V/7-AAD assay and percentage of cell death was plotted. Data are mean ± SD (*n* = 3). ^*^P < 0.05, ^**^P < 0.01, ^***^P < 0.001 versus nontreated control; ^*#*^*P* < 0.05, ^*##*^*P* < 0.01, ^*###*^*P* < 0.001 versus ouabain-treated LSCs at the same concentration; two-sided Student’s *t* test

AML patient-derived primary cells were obtained from PBMCs of relapsed patients and were similarly treated with ouabain (0 − 100 nM) for 48 h. The majority of primary AML Pr3R (~ 95%) cells in StemSpan culture were LSCs, while Pr2R cells contained mainly LSCs (~ 40%) and LBs (~ 50%) (Additional file 1: Table S2). Consistent with the findings obtained from human AML cell lines, we observed that Pr3R cells were very responsive to ouabain (LC50 value of 24.92 nM), similarly to those of primitive KG-1 and Kasumi-3 cells (Fig. 5A, left). Remarkably, we validated in Pr2R cells that LSCs were more sensitive to ouabain than its counterpart LBs (Fig. 5A, right), thus strengthening that ouabain efficiently targets LSCs.

To further support the potential clinical application of ouabain, its cytotoxicity was also evaluated in normal PBMCs in a concentration range of 0 − 500 nM for up to 72 h, which covered the cytotoxic concentrations of the tested AML cells. Additional file 1: Fig. S5 shows that ouabain had no significant cytotoxic effect on normal PBMCs at a similar dosing condition as the tested AML cells. For example, the cytotoxic concentrations of ouabain were above 200 nM at 48 h (LC50 not available) versus the highest LC50 of the tested AML cells in HL-60 at 46.35 nM, indicating that the pharmacologic concentrations of ouabain selectively kill AML cells, while sparing non-malignant PBMCs. Although normal CD34^+^ HSPCs accounts for less than 0.05% and 1% of total cells in peripheral blood and bone marrow, respectively [38], we tested and confirmed that ouabain is remarkably less toxic against enriched CD34^+^ HSPCs from cord blood and G-CSF-mobilized peripheral blood when compared to enriched LSCs from KG-1 cells (Fig. 5B).

### Cytotoxicity of ouabain is in part cell cycle dependent

**Fig. 6:**
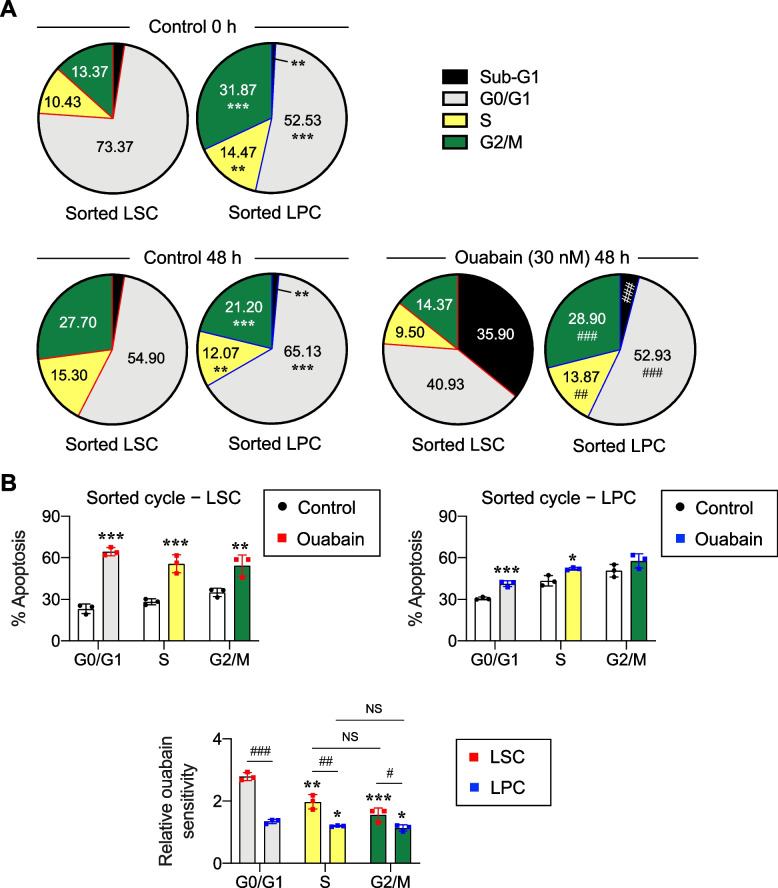
LSCs exhibit greater ouabain sensitivity than LPCs regardless of cell cycle status. **A** Cell cycle distribution of enriched LSCs and LPCs with or without ouabain treatment (30 nM) as analyzed by flow cytometry using PI DNA dye at 0 − 48 h. Data are mean (*n* = 3). ^**^P < 0.01, ^***^P < 0.001 versus nontreated LSCs in the same phase of cell cycle; ^##^*P* < 0.01, ^###^*P* < 0.001 versus ouabain-treated LSCs in the same phase of cell cycle; two-sided Student’s *t* test. **B** Enriched LSCs and LPCs were sorted according to cell cycle distribution analyzed by flow cytometry using Hoechst 33342 DNA dye into G0/G1, S, and G2/M cells and subsequently treated with ouabain (30 nM) for 48 h. (upper) Percentage of apoptosis in cells obtained from each phase of cell cycle in LSCs (left) and LPCs (right) was determined by Annexin V/7-AAD assay. Data are mean (*n* = 3). ^**^P < 0.01, ^***^P < 0.001 versus nontreated control in the same phase of cell cycle in LSCs or LPCs; two-sided Student’s *t* test. (lower) Relative ouabain sensitivity was calculated from the ratio of apoptosis of treated to nontreated control cells. Data are mean (*n* = 3). ^*^P < 0.05, ^**^P < 0.01, ^***^P < 0.001 versus nontreated control in G0/G1 cells in LSCs or LPCs; one-way ANOVA with Tukey’s posttest. ^#^*P* < 0.05, ^##^*P* < 0.01, ^###^*P* < 0.001 versus ouabain-treated LSCs in the same phase of cell cycle; two-sided Student’s *t* test. NS, not significant

It is well accepted that cell cycle has an important influence on tumor cell sensitivity to anticancer agents, depending on the mechanism of action [39]. For example, cytarabine and 5-fluorouracil that are antimetabolites are most effective during the S phase, while taxoids that stabilize microtubules are most potent during the M phase [40]. To investigate whether cell cycle contributes to the difference in ouabain sensitivity between LSCs and LPCs, we first performed flow cytometry-based cell cycle analysis in enriched LSCs and LPCs in the presence or absence of ouabain. Cell cycle profile of LSCs at 24 h post-starvation (at the time of treatment, 0 h) showed a greater percentage of cells in G0/G1 phase than LPCs, while LPCs were shown to have more cells distributed in S and G2/M phases (Fig. 6A), suggesting that LSCs were more quiescent than LPCs. Ouabain treatment at 30 nM for 48 h resulted in a remarkable increase in sub-G0 cells in LSCs (35.90% versus 2.63% in nontreated control; *P* = 0.0002), while it had minimal effect in LPCs (4.07% versus 1.47% in nontreated control; *P* = 0.0034), thereby confirming that ouabain significantly induced apoptosis in LSCs and that its effect was more pronounced in LSCs than LPCs.

Next, we evaluated whether different phases of cell cycle in LSCs and LPCs respond to ouabain differently. Enriched LSCs and LPCs were similarly analyzed for cell cycle distribution using flow cytometry and subsequently sorted into G0/G1, S, and G2/M cell fractions. Then, enriched G0/G1, S, and G2/M cells were treated with ouabain for 48 h and apoptosis was evaluated by Annexin V/7-AAD assay. Figure 6B (upper) shows that ouabain (30 nM) remarkably induced apoptosis in all phases of cell cycle in LSCs when compared to its counterpart control at the same cycle phase. In LPCs, ouabain (30 nM) slightly induced apoptosis in G0/G1 and S cells but not in G2/M cells. Hence, the relatively lower sensitivity of LPCs to ouabain may be in part due to the residing of a substantial proportion (~ 30%) of LPCs in G2/M phase. To better compare the ouabain sensitivity in each cell cycle phase of LSCs and LPCs, relative drug sensitivity was calculated from the ratio of apoptosis of treated to their counterpart nontreated cells. The results confirmed that LSCs were more sensitive to ouabain than LPCs in all phases of cell cycle (Fig. 6B, lower). Interestingly, G0/G1 cells were the most responsive among different phases of LSCs and LPCs, indicating that the cytotoxicity of ouabain is in part cell cycle dependent through no known mechanism. Herein, we can also imply that the difference in ouabain sensitivity between LSCs and LPCs was in part due to their differences in cell cycle distribution and intrinsic apoptosis regulatory mechanisms.

### Ouabain induces both intrinsic and extrinsic pathways of apoptosis

**Fig. 7:**
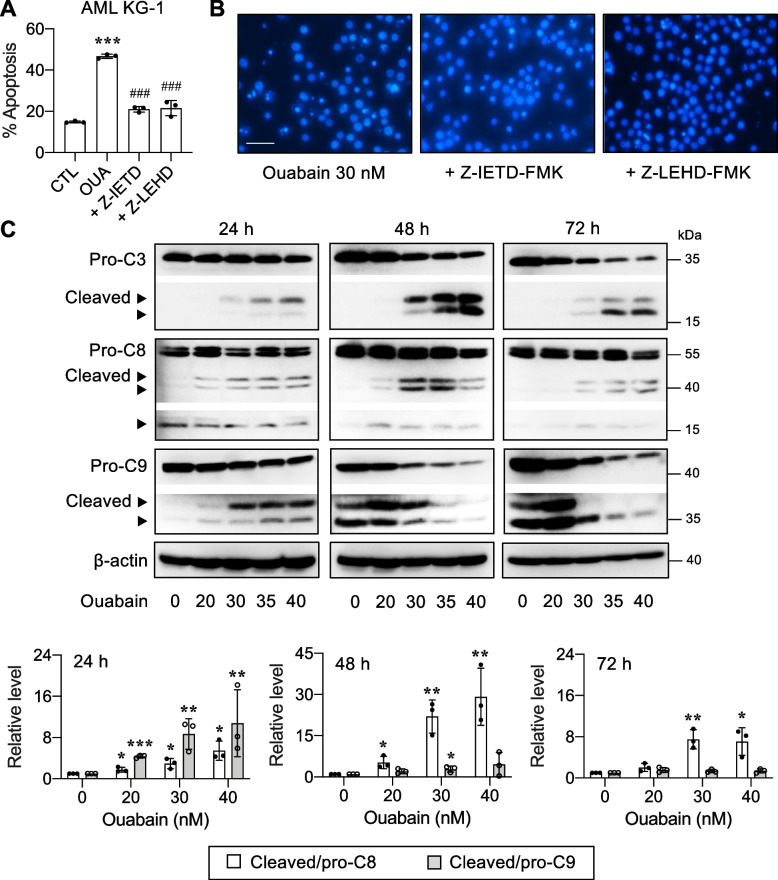
Ouabain induces caspase-dependent apoptosis in AML cells. AML KG-1 cells were treated with ouabain (30 nM) in the presence or absence of caspase 8 inhibitor zVAD-IETD (10 μM) and caspase-9 inhibitor zVAD-LEHD (10 μM) for 48 h. **A** Percentage of apoptosis as evaluated by Annexin V/7-AAD assay. **B** Representative micrographs of Hoechst 33342 nuclear staining under an inverted fluorescence microscope. Condensed and/or fragmented nuclei were considered apoptotic cells. Scale bar = 100 μm. **C** Western blot analysis of pro- and cleaved (active) caspase-3 (C3), caspase-8 (C8), and caspase-9 (C9) levels upon ouabain treatment (0 − 40 nM) for 24 − 72 h. β-actin was used as a loading control. Quantitative analysis of caspase activation as a ratio of cleaved to pro-C8 and C9 levels by densitometry is shown (see also Additional file 1: Fig. S6). Data are mean ± SD (*n* = 3). ^*^P < 0.05, ^**^P < 0.01, ^***^P < 0.001 versus nontreated control cells; two-sided Student's *t* test

Caspase-9 serves as the apical caspase of the intrinsic apoptosis pathway, while caspase-8 represents the apical caspase of the extrinsic pathway [41, 42]. To characterize the mechanisms underlying apoptotic response to ouabain in AML cells, KG-1 cells were first treated with ouabain (30 nM) in the presence or absence of caspase-8 inhibitor zVAD-IETD (10 μM) and caspase-9 inhibitor zVAD-LEHD (10 μM), and apoptosis was evaluated by Annexin V/7-AAD and Hoechst 33342 assays at 48 h. Figure 7A and B shows that both caspase-8 and 9 inhibitors effectively inhibited apoptosis induced by ouabain in KG-1 cells, indicating that caspase-dependent pathways play essential roles in ouabain-induced apoptosis and that both extrinsic and intrinsic death pathways are involved in the process. Western blot analysis showed a dose- and time-dependent increase in caspase-3 activation (cleaved/pro-caspase) by ouabain (Fig. 7C and Additional file 1: Fig. S6A). Caspase-9 was found to be highly activated in a dose-dependent manner at 24 h and gradually deactivated at 48 − 72 h, likely due to the massive loss of pro-caspase-9 (Additional file 1: Fig. S6B), while caspase-8 remained activated throughout the time of the experiment, thereby strengthening that ouabain induces both intrinsic and extrinsic pathways of apoptosis.

**Fig. 8:**
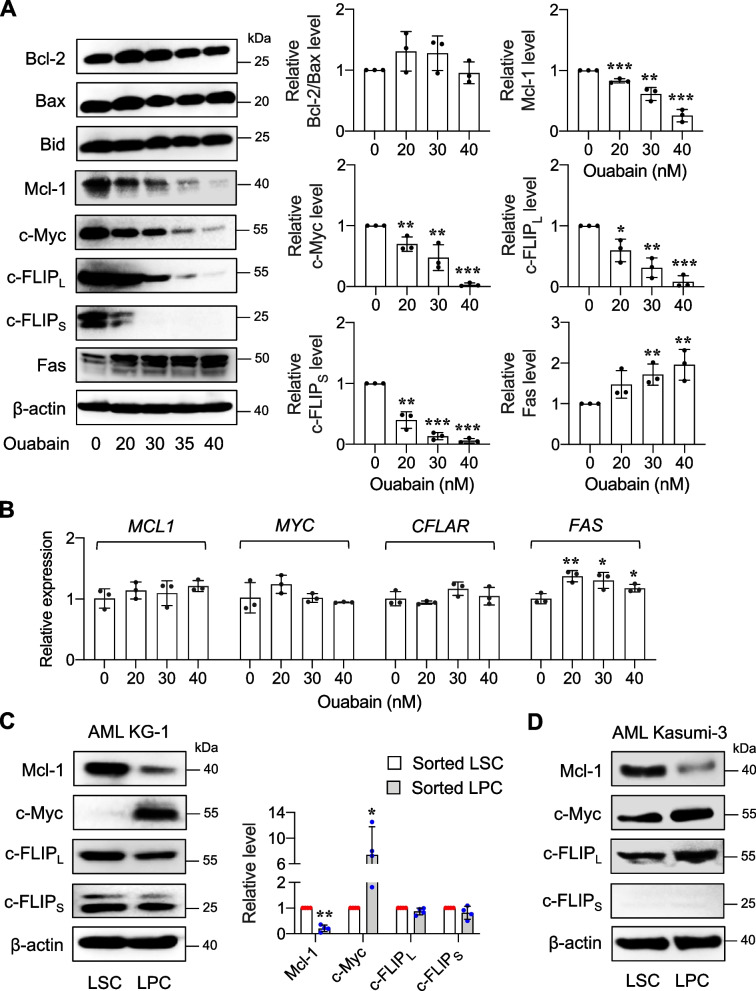
Ouabain modulates key apoptosis regulatory proteins in both intrinsic and extrinsic pathways of apoptosis in AML cells. **A**, **B** AML KG-1 cells were treated with ouabain (0 − 40 nM) for 48 h. **A** Western blot analysis of the key apoptosis regulatory proteins, including Bcl-2, Bax, Bid, Mcl-1, c-Myc, c-FLIP, and Fas. β-actin was used as a loading control. Densitometric analysis of Bcl-2 and Bax presented as a ratio of Bcl-2 to Bax, Mcl-1, c-Myc, c-FLIP_L_, c-FLIP_S,_ and Fas levels is shown. Data are mean ± SD (*n* = 3). ^*^P < 0.05, ^**^P < 0.01, ^***^P < 0.001 versus nontreated control cells; two-sided Student's *t* test. **B** qPCR of *MCL1*, *MYC*, *CFLAR* (encoding c-FLIP), and *FAS* mRNA expression. *GAPDH* was used as a house-keeping gene. Data are mean ± SD (*n* = 3). ^*^P < 0.05, ^**^P < 0.01, ^***^P < 0.001 versus nontreated control cells; two-sided Student's *t* test. **C**, **D** Western blot analysis of Mcl-1, c-Myc, c-FLIP_L_, and c-FLIP_S_ in enriched LSCs and LPCs from AML KG-1 (**C**) and Kasumi-3 (**D**) cells. Data are mean ± SD (*n* = 4). ^*^P < 0.05, ^**^P < 0.01 versus enriched LSCs; two-sided Student's *t* test

To elucidate the underlying mechanisms of ouabain-mediated apoptosis, we monitored the levels of key apoptosis regulatory proteins in the intrinsic and extrinsic pathways, including Bcl-2, Bax, Mcl-1, Bid, c-Myc, c-FLIP (cellular FLICE inhibitory protein), and Fas, following ouabain treatment for 48 h. The results showed that the ratio of antiapoptotic Bcl-2 to proapoptotic Bax, which generally determines the susceptibility of a cell to apoptosis [43], was relatively unchanged (Fig. 8A), suggesting their nondominant role in ouabain cytotoxicity. Despite being a known molecule that links the two apoptosis pathways [44], the level of Bid was not significantly changed, and its cleaved form (truncated Bid, tBid) was undetectable, suggesting its unlikely involvement in the coactivation of both pathways herein. Remarkably, we observed a dose-dependent decrease in Mcl-1, c-Myc, and c-FLIP in both long (c-FLIP_L_) and short (c-FLIP_S_) isoforms as opposed to a dose-dependent increase in Fas receptor in ouabain-treated cells. To substantiate whether these changes were seen at the transcriptional level, mRNA expression of *MCL1*, *MYC*, *CFLAR* (encoding c-FLIP), and *FAS* in response to ouabain was evaluated by qPCR analysis. A significant upregulation of *FAS* was observed in ouabain-treated cells (Fig. 8B), suggesting that the induction of Fas was in part through a transcription-dependent program. By contrast, ouabain caused a minimal change in *MCL1*, *MYC*, and *CFLAR*, despite a substantial decrease in protein levels, indicating that its action on Mcl-1, c-Myc, c-FLIP_L_, and c-FLIP_S_ is merely at the posttranscriptional level.

Having demonstrated the differential cytotoxic effects of ouabain against LSCs and LPCs, we profiled the basal levels of Mcl-1, c-Myc, c-FLIP_L,_ and c-FLIP_S_ in the enriched LSCs and LPCs. Figure 8C shows that while the levels of c-FLIP_L_ and c-FLIP_S_ were comparable in LSCs and LPCs from AML KG-1 cells, LSCs had a relatively higher level of Mcl-1 and a lower level of c-Myc. Similar findings on the Mcl-1 and c-Myc levels were observed in the enriched LSCs and LPCs from AML Kasumi-3 cells, though the c-FLIP_S_ was barely detectable (Fig. 8D). Altogether, these results suggested that Mcl-1 and c-Myc may be important regulators of ouabain sensitivity in AML cells.

### Mcl-1 and c-Myc mediate ouabain-induced apoptosis

**Fig. 9:**
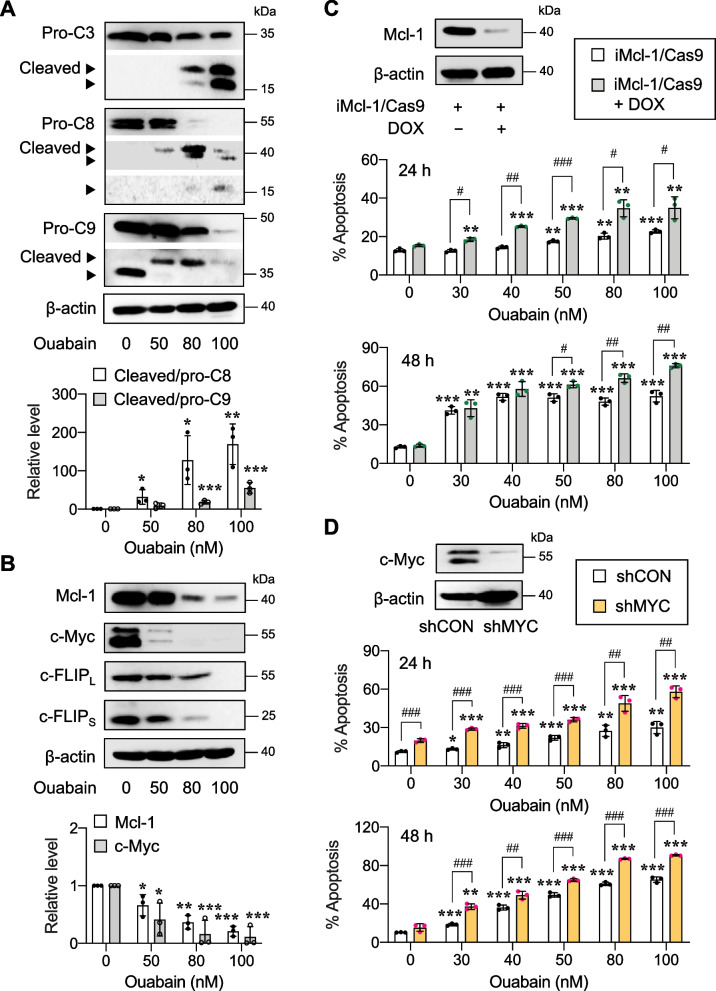
Mcl-1 and c-Myc are key mediators of ouabain-indued apoptosis in AML cells. **A**, **B** AML HL-60 cells were treated with ouabain (0 − 100 nM) for 48 h. **A** Western blot analysis of pro- and cleaved (active) caspase-3 (C3), caspase-8 (C8), and caspase-9 (C9) levels. Quantitative analysis of caspase activation as a ratio of cleaved to pro-C8 and C9 levels by densitometry is shown. Data are mean ± SD (*n* = 3). ^*^P < 0.05, ^**^P < 0.01, ^***^P < 0.001 versus nontreated control cells; two-sided Student's *t* test. **B** Western blot analysis of the key apoptosis regulatory proteins, including Mcl-1, c-Myc, c-FLIP_L_, and c-FLIP_S_, and their densitometric analysis. β-actin was used as a loading control. Data are mean ± SD (*n* = 3). ^*^P < 0.05, ^**^P < 0.01, ^***^P < 0.001 versus nontreated control cells; two-sided Student's *t* test. **C** HL-60 cells were genetically knocked down with sgRNA sequence against *MCL1* in the DOX-inducible CRISPR/Cas9 system. (upper) Western blot analysis showing Mcl-1 knockdown efficiency in iMcl-1/Cas9 cells after DOX treatment (1 μg/mL). (lower) Percentage of apoptosis as evaluated by Annexin V/7-AAD assay following ouabain treatment (0 − 100 nM) with or without DOX for 24 − 48 h. Data are mean (*n* = 3). ^*^P < 0.05, ^**^P < 0.01, ^***^P < 0.001 versus nontreated control cells; ^#^*P* < 0.05, ^##^*P* < 0.01, ^###^*P* < 0.001 versus ouabain-treated iMcl-1/Cas9 cells without DOX; two-sided Student’s *t* test. **D** Depletion of c-Myc was performed in HL-60 cells using shRNA sequence against *MYC*. (upper) Western blot analysis showing c-Myc knockdown efficiency in shMYC cells. (lower) Percentage of apoptosis as evaluated by Annexin V/7-AAD assay following ouabain treatment (0 − 100 nM) for 24 − 48 h. Data are mean (*n* = 3). ^*^P < 0.05, ^**^P < 0.01, ^***^P < 0.001 versus nontreated control cells; ^##^*P* < 0.01, ^###^*P* < 0.001 versus ouabain-treated shCON cells; two-sided Student’s *t* test

Next, we manipulated Mcl-1 and c-Myc to ascertain whether they indeed regulated the cytotoxicity of ouabain in AML cells. Due to limitations in the efficiency of gene knockdown, either CRISPR/Cas9 or shRNA, in AML KG-1 cells, which are the early myeloblasts that are notoriously difficult to transfect similarly to CD34^+^ HSPCs, experiments were performed in AML HL-60 cells, which are more mature. In HL-60 cells, we first checked and confirmed that the profiles of key apoptosis proteins in response to ouabain were similar to those of KG-1 cells. Figure 9A and B shows that ouabain similarly activated both caspase-8 and caspase-9 in a dose-dependent manner at 48 h in concomitant with a dose-dependent decrease in the identified key antiapoptotic proteins, including Mcl-1, c-Myc, c-FLIP_L_, and c-FLIP_S_, though the effects on c-FLIP_L_ and c-FLIP_S_ occurred at relatively higher doses (> 50 nM) (see also Additional file 1: Fig. S7 for additional proteins). CRISPR/Cas9 targeting human *MCL1* and shRNA against human *MYC* were used to repress Mcl-1 and c-Myc in HL-60 cells, respectively, and Western blotting was performed to validate the decreased protein level prior to experiments. Depletion of Mcl-1 and c-Myc sensitized HL-60 cells to ouabain-induced apoptosis (Fig. 9C and D), highlighting the contribution of Mcl-1 and c-Myc to ouabain sensitivity in AML cells.

### LSCs and LPCs exhibit different apoptotic machinery in response to ouabain

**Fig. 10:**
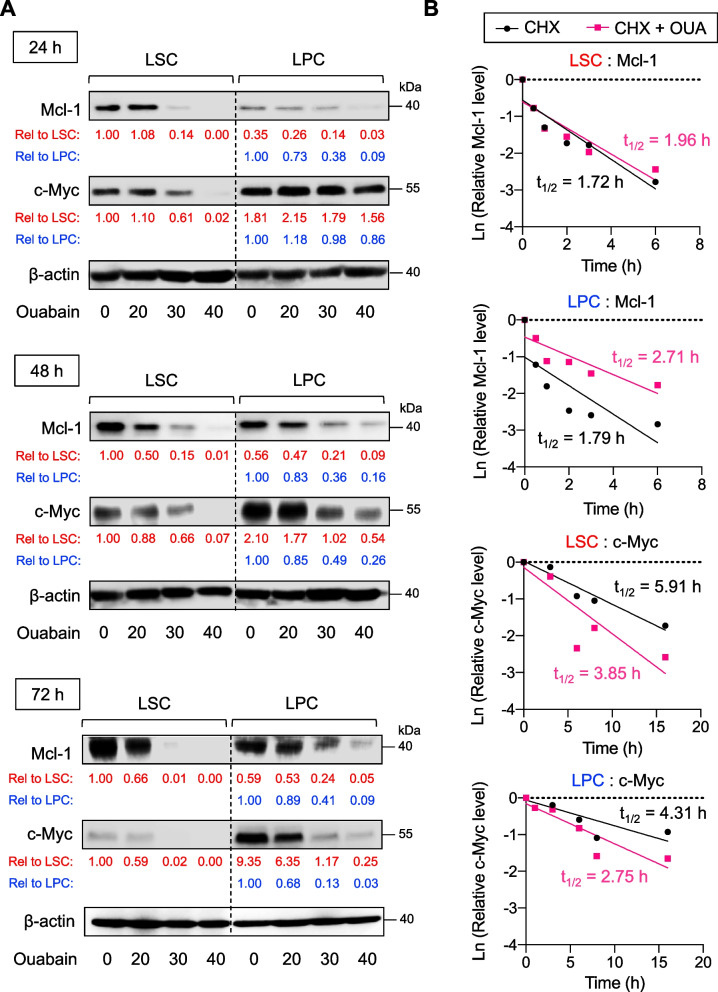
Ouabain induces a rapid loss of Mcl-1 and c-Myc in LSCs. **A** Kinetic analysis of Mcl-1 and c-Myc in enriched LSCs and LPCs from AML KG-1 cells following ouabain treatment (0 − 40 nM) for 24 − 72 h by Western blotting. β-actin was used as a loading control. Quantitative analysis of Mcl-1 and c-Myc levels is shown under the immunoblots. Dash line indicates juxtapose lanes taken from the same blots. **B** The half-life (t_1/2_) of Mcl-1 and c-Myc in enriched LSCs and LPCs upon ouabain treatment (40 nM) was determined in the presence of the protein synthesis inhibitor CHX (10 μg/mL)

The question remains as to why LSCs appeared to be more sensitive to ouabain than LPCs. To address this, we performed time course and dose–response experiments to evaluate the effects of ouabain on Mcl-1 and c-Myc kinetics in LSCs and LPCs in study model 2 using Western blotting. Figure 10A demonstrates that although ouabain similarly reduced Mcl-1 and c-Myc in a dose- and time-dependent manner in both LSCs and LPCs, its actions appeared to be more rapid in LSCs. For instance, at 24 h, ouabain (30 nM) induced Mcl-1 reduction by approximately 85% in LSCs versus 60% in LPCs. The greater effect of ouabain on Mcl-1 in LSCs relative to LPCs remained at 48 and 72 h—85% versus 65% reduction at 48 h and nearly 100% versus 60% reduction at 72 h. For c-Myc, the effect of ouabain (30 nM) was first observed at 24 h and only in LSCs but not in LPCs. Then at 72 h, the degree of c-Myc reduction induced by ouabain (30 nM) was comparable between LSCs and LPCs. Likewise, the inhibitory effects of ouabain on c-FLIP_L_ and c-FLIP_S_ were also much faster and greater in LSCs than LPCs at 24 h and were comparable at 48 and 72 h (Additional file 1: Fig. S8). Altogether, our data suggest the delayed responses of key apoptosis regulatory proteins to ouabain in LPCs that could be associated with its delayed apoptotic response when compared to LSCs. Interestingly, ouabain exerted a relatively lower effect on these proteins, particularly Mcl-1, in LPCs even at the longer exposure of 72 h.

Mcl-1 and c-Myc protein half-lives in the presence or absence of ouabain were further compared in LSCs and LPCs using the CHX-chase assay. CHX inhibited new protein synthesis, thereby decreasing Mcl-1 and c-Myc levels over time by the degradation machinery even when the cells were left untreated. Ouabain (40 nM) remarkably accelerated c-Myc degradation in LSCs and LPCs, with an approximately 40% reduction in the c-Myc half-life (Fig. 10B). For Mcl-1, which was a very short-lived protein (t_1/2_ < 2 h in LSCs and LPCs), we surprisingly found that ouabain (40 nM) had minimal effect on the Mcl-1 half-life in LSCs and even increased the half-life in LPCs, despite its remarkable decrease at the protein level at 24 − 72 h in both cells in the absence of CHX (Fig. 10A). These data indicate that ouabain-mediated Mcl-1 depletion was not directly associated with protein degradation, but rather with new protein synthesis. Although the regulatory mechanisms of Mcl-1 by ouabain are still unclear herein, the distinct effects of ouabain on Mcl-1 in LSCs and LPCs may be a key to their distinct responses. Notably, a direct inhibition of Mcl-1 by a small molecule inhibitor Mcl-1 inhibitor II caused remarkable apoptosis in both LSCs and LPCs in a dose-dependent manner, thus confirming that Mcl-1 is essential for LSC and LPC survival (Additional file 1: Fig. S9).

## Discussion

In the past decades, the potential of cardiac glycosides as anticancer agents has been evaluated in various solid tumors and hematologic malignancies in multiple preclinical and clinical studies, originally arising from the epidemiological evidence of a lower risk of certain cancers in patients under treatment with cardiac glycosides [15, 45]. Recent technologies, e.g., in silico analysis and high-throughput screening, oftentimes predicted cardiac glycosides as potential candidates for repurposing for cancer therapeutics [14, 46, 47]. However, none of these studies focused on the uses of cardiac glycosides for targeting LSCs and identified the mechanisms of action underlying their superior sensitivity, which could be important for their future clinical application and for the design of novel treatment strategies for AML. Here, we reported that nanomolar concentrations of cardiac glycosides induced apoptosis in various AML cell lines, with ouabain being the most potent cardiac glycoside tested, and KG-1 and Kasumi-3 cells, which are early, CD34^+^ myeloblasts, being the most responsive cells tested (Figs. 1 and 2), suggesting the strong cytotoxic effects of cardiac glycosides against primitive AML cells. The user-designed D6-MA was proven to be much more potent than digoxin, which was not very effective against the relatively more mature AML M2 and M3 cells at nanomolar concentrations when compared using LC50. However, ouabain showed approximately two-fold greater potency relative to D6-MA in all AML cell lines tested with minimal cytotoxic effect on normal PBMCs (Additional file 1: Fig. S5).

One common hallmark that is shared across all AML subtypes is differentiation blockage, causing an accumulation of immature malignant leukemic myeloblasts [22]. Differentiation therapy, i.e., all-trans retinoic acid (ATRA) and arsenic trioxide, has been shown to be an effective treatment for acute promyelocytic leukemia (APL), a distinct AML subtype characterized by the expression of the PML/RARA fusion protein [23]. Previous studies reported that the induction of AML differentiation could sometimes be accompanied by apoptosis [36, 37, 48]. Herein, we established two study models to specifically determine the effects of ouabain on LSC and LPC death and differentiation using AML KG-1 cells. The major difference between the two models is that model 1 allowed dynamic changes between LSCs and LPCs, while LSCs could not be further differentiated in study model 2. Using study model 1, we found that ouabain could induce the differentiation of LSCs into LPCs, but this effect was much lower than its apoptotic effect given at the same dose (Fig. 3), suggesting that the cytotoxicity of ouabain in primitive AML cells may not be directly associated with the induction of cell differentiation. Notably, as we detected the differentiation only in viable cells, it is not surprising that such effect of ouabain at 30 − 40 nM was lowest at 72 h, when the majority of the cells underwent apoptosis. Using study model 2, we were able to make a side-by-side comparison of the cytotoxic effect of ouabain in LSCs and LPCs. The results showed that ouabain preferentially induces cell death in LSCs when compared to LPCs, independent of the cell differentiation status (Fig. 4). To support this notion, ouabain is further validated to effectively target LSCs in primary AML cells obtained from relapsed patients (Fig. 5A).

The responses of tumor cells to various chemotherapy and targeted agents are largely dependent on the cell cycle state [39, 40]. Previous studies reported that cell cycle quiescence (G0 phase) underlay the chemotherapy resistance of LSCs, leading to AML relapse [49, 50], and their induction into cell cycle entry was shown to sensitize these LSCs to cytarabine in vivo [51]. Analysis of cell cycle distribution and subsequent cell sorting into different phases of cell cycle in LSCs and LPCs confirmed that the majority of LSCs were in G0/G1 phase and revealed for the first time that G0/G1 cells in both LSCs and LPCs were relatively more responsive to ouabain than other phases of cell cycle (Fig. 6). Interestingly, LSCs had higher ouabain sensitivity than its counterpart LPCs in all phases of cell cycles, ranking G0/G1 LSCs as the highest ouabain responders among all AML subpopulations, and suggesting that the difference in ouabain sensitivity between LSCs and LPCs was in part due to their differences in cell cycle distribution and intrinsic apoptosis regulatory mechanisms. As chemotherapy, including the S-phase-specific cytarabine that is a core drug for AML therapy, is not very effective against G0/G1 cells in general [40, 50], our findings on the high ouabain sensitivity of G0/G1 cells, particularly those in LSCs, strongly supported its application for AML therapy.

Apoptosis was the major mode of cell death induced by ouabain and other cardiac glycosides via caspase-dependent intrinsic and extrinsic pathways of apoptosis (Additional file 1: Fig. S3 and Fig. 7). Profiling of various key apoptosis-regulatory proteins in the intrinsic and extrinsic pathways and the subsequent comparison of those ouabain target proteins between LSCs and LPCs suggested that Mcl-1 and c-Myc may be important regulators of ouabain sensitivity in AML cells that plausibly caused the distinct apoptotic responses between LSCs and LPCs (Fig. 8), although we did not exclude the possible roles of c-FLIP in both isoforms herein. Using CRISPR/Cas9 and shRNA-mediated gene knockdown, we validated the relevance of Mcl-1 and c-Myc downregulation to ouabain-induced apoptosis in AML cells (Fig. 9). Mcl-1 is an antiapoptotic protein of the Bcl-2 family that has been listed as a common target of cardiac glycoside UNBS1450 in a panel of tumor cells of different origins, e.g., lung, prostate, breast, blood and nerve tissue [52]. To support the generality of cardiac glycoside effects on Mcl-1, our group previously showed that ouabain, digitoxin, and the user-designed D6-MA induced Mcl-1 degradation in NSCLC cell lines under attached and detached conditions via the ubiquitin-proteosome proteolytic pathway [20, 21, 53]. Interestingly, a similar mechanism involving Mcl-1 stability and proteasome mediated by cardiac glycosides was not observed in AML cells in the present study—we found that ouabain had a minimal effect on the Mcl-1 half-life despite its enormous induction of Mcl-1 depletion. As ouabain also had a minimal effect on the *MCL1* gene (Fig. 8), we postulated that ouabain may interfere with new Mcl-1 protein synthesis in AML cells at translation initiation, which requires further study. To support this notion, cardiac glycosides, i.e., digoxin, digoxigenin, digitoxigenin, and lanatoside C, have been shown to be potential inhibitors of eIF4A-mediated translation [54]. eIF4A is one of the three core components of the eukaryotic translation initiation factor (eIF) 4F complex, which assists in the recruitment of the 43S pre-initiation complex (PIC) to mRNA to initiate the translation. Mcl-1 is a highly unstable protein that requires active translation to maintain its level [55], and it is therefore not surprising that Mcl-1 is among many oncoproteins that is dependent on the helicase activity of eIF4A for its expression [56]. The rapid loss of Mcl-1 by ouabain was observed in LSCs, which expressed high Mcl-1, and such effect was greater than that in LPCs even at the longer exposure of 72 h (Figs. 8 and 10), supporting that the preference of ouabain in targeting LSCs was in part associated with Mcl-1.

c-Myc is an oncogene that is aberrantly expressed, e.g., from *MYC* amplification and chromosomal translocation, in the majority (> 70%) of human cancers [57]. More than 90% of AML cases were shown to have high c-Myc level in bone marrow when compared with normal bone marrow, as evaluated by immunohistochemistry [58]. Here, we found that ouabain induced c-Myc downregulation in AML cells, which underlay apoptosis, by reducing its protein stability, independent of the transcriptional modulation (Figs. 8, 9 and 10). The effects of cardiac glycosides, i.e., digitoxin, cymarin, somalin, and proscillaridin A, on c-Myc inhibition have been previously reported elsewhere [13, 59, 60]. Herein, we extend the knowledge that ouabain could reduce the c-Myc protein half-life in both LSCs and LPCs by approximately 40% (Fig. 10B). Notably, we could not rule out the possibility that ouabain may additionally inhibit c-Myc via eIF4A-mediated translation, as c-Myc is one of the reported oncoprotein targets of eIF4A [56]. Remarkably, even though the basal level of c-Myc in LPCs was much greater than in LSCs, ouabain’s effect on c-Myc in LSCs was greater than that in LPCs, particularly at 24 h, strengthening that ouabain preferentially targets LSCs through distinct intrinsic apoptosis machinery.

## Conclusion

Our data demonstrate that ouabain at nanomolar concentrations preferentially targets LSCs, yet is very effective against LPCs, and induces apoptosis without a need of undergoing cell differentiation. We revealed for the first time that the distinct cell cycle distribution and intrinsic apoptotic machinery in LSCs and LPCs are attributed to this differential ouabain sensitivity. G0/G1 cells in LSCs were the top ouabain responders among all AML subpopulations. Mcl-1 and c-Myc were the key mediators that determined ouabain sensitivity in AML cells. We further unveiled that ouabain induced the more rapid loss of Mcl-1 and c-Myc in LSCs, which could explain its preferential sensitivity. Further studies are required to investigate the association of ouabain and translation initiation, particularly those that involve Mcl-1 and c-Myc regulation. Overall, our findings support that ouabain has promising potential to be repurposed as an anticancer agent that targets LSCs and open avenues for further development of other cardiac glycosides or synthetic analogs with greater potency against LSCs to control its self-renewal for the treatment of relapsed/refractory AML.

### Supplementary Information

**Additional file 1: Table S1.** Clinical characteristics of AML patient-derived primary cells. **Table S2.** Profiles of different AML subpopulations at the time of isolation and in culture. **Fig. S1.** Purity of CD34^+^ HSPCs isolated from human cord blood and G-CSF-mobilized peripheral blood. **Fig. S2.** TIM3 expression in LSCs and normal HSPCs. **Fig. S3.** Apoptosis is a major mode of cell death induced by cardiac glycosides in AML cells. **Fig. S4.** Percentage of CD15^+^ blasts in live (Annexin V^−^) cells upon ouabain treatment in study model 2. **Fig. S5.** Ouabain had minimal effect on normal PBMCs. **Fig. S6.** Ouabain induces caspase activation in AML cells. **Fig. S7.** Profiles of key apoptosis regulatory proteins in response to ouabain in AML HL-60 cells. **Fig. S8.** Ouabain induces a rapid loss of c-FLIP_L_ and c-FLIP_S_ in LSCs. **Fig. S9.** Small molecule inhibition of Mcl-1 similarly caused apoptosis in LSCs and LPCs.

## Data Availability

The datasets used and/or analyzed during the current study are available from the corresponding authors on reasonable request.

## Abbreviations

AML: Acute myeloid leukemia
APL: Acute promyelocytic leukemia
ATCC: American Type Culture Collection
ATRA: All-trans retinoic acid
C3: Caspase-3
C8: Caspase-8
C9: Caspase-9
c-FLIP: Cellular FLICE inhibitory protein
CHX: Cycloheximide
D6-MA: Digitoxigenin-α-L-rhamnoside
DOX: Doxycycline
eIF: Eukaryotic translation initiation factor
FACS: Fluorescence activated cell sorting
HBr: Hexadimethrine bromide
JCRB: Japanese Collection of Research Bioresources
LB: Leukemic blast
LC50: Lethal concentration 50
LPC: Leukemic progenitor cell
LSC: Leukemic stem cell
MC: Methylcellulose
NSCLC: Non-small cell lung cancer
PBMC: Peripheral blood mononuclear cell
PI: Propidium iodide
PIC: Pre-initiation complex
sgRNA: Single guided RNA
shRNA: Short hairpin RNA
t_1/2_: Half-life
