# Supplementary material for: Cardiac glycoside ouabain efficiently targets leukemic stem cell apoptotic machinery independent of cell differentiation status
Source: Cell Commun Signal. 2023 Oct 12;21:283. doi: 10.1186/s12964-023-01317-8 (PMC10568939; doi:10.1186/s12964-023-01317-8)
Supplement: Supplementary file 2 — Additional file 1: Table S1. Clinical characteristics of AML patient-derived primary cells. Table S2. Profiles of different AML subpopulations at the time of isolation and in culture. Fig. S1. Purity of CD34+ HSPCs isolated from human cord blood and G-CSF-mobilized peripheral blood. Fig. S2. TIM3 expression in LSCs and normal HSPCs. Fig. S3. Apoptosis is a major mode of cell death induced by cardiac glycosides in AML cells. Fig. S4. Percentage of CD15+ blasts in live (Annexin V−) cells upon ouabain treatment in study model 2. Fig. S5. Ouabain had minimal effect on normal PBMCs. Fig. S6. Ouabain induces caspase activation in AML cells. Fig. S7. Profiles of key apoptosis regulatory proteins in response to ouabain in AML HL-60 cells. Fig. S8. Ouabain induces a rapid loss of c-FLIPL and c-FLIPS in LSCs. Fig. S9. Small molecule inhibition of Mcl-1 similarly caused apoptosis in LSCs and LPCs. [file 12964_2023_1317_MOESM1_ESM.pdf]

Supplementary information

Additional file 1: Supplementary Table S1 and S2 and Figs. S1−S9.

Cardiac glycoside ouabain efficiently targets leukemic stem cell apoptotic machinery independent of cell differentiation status

Jirarat Poohadsuan[1], George A. O'Doherty[2], Weerapat Owattanapanich[3,4], Smith Kungwankiattichai[3,4], Yon Rojanasakul[5,6], Surapol Issaragrisil[1,3] and Sudjit Luanpitpong[1,*]

[1]Siriraj Center of Excellence for Stem Cell Research, Faculty of Medicine Siriraj Hospital, Mahidol University, Bangkok, Thailand; [2]Department of Chemistry and Chemical Biology, Northeastern University, Boston, MA, USA; [3]Division of Hematology, Department of Medicine, Faculty of Medicine Siriraj Hospital, Mahidol University, Bangkok, Thailand; [4]Center of Excellence of Siriraj Adult Acute Myeloid/Lymphoblastic Leukemia, Faculty of Medicine Siriraj Hospital, Mahidol University, Bangkok, Thailand; [5]Department of Pharmaceutical Sciences, West Virginia University, Morgantown, WV, USA; [6]WVU Cancer Institute, West Virginia University, Morgantown, WV, USA.

Correspondence: Sudjit Luanpitpong, Siriraj Center of Excellence for Stem Cell Research, Faculty of Medicine Siriraj Hospital, Mahidol University, 2 Siriraj Hospital, Bangkoknoi, Bangkok 10700, Thailand; Tel.: +66 2 419 2907; Email: suidjit@gmail.com.

Table S1 Clinical characteristics of AML patient-derived primary cells.

DES	PROG	FAB	Age (yr)	Sex	Immunophenotyping	Cytogenetics	Molecular analyses	% blasts in PB	WBC count
Pr2R	Relapse	M2	65	M	$CD34^+$ $CD13^+$ $CD33^+$ $CD117^+$ MPO^+ HLA-DR^+	46, XY, t(8;21)(q21;q22), del(9)(q22q34) [17]/ 46, XY [3]	FLT3 D635V, JAK2 V617F	34%	16300/μL
Pr3R	Relapse	M0	78	F	$CD34^+$ $CD13^+$ $CD33^+$ $CD117^+$ MPO^- HLA-DR^+	46, XX, del(5)(q15q33) [10]/ 46, XX [10]	SF3B1 K700E, TET2 R1572Q, RUNX1 T181P	36%	7800/μL

Abbreviations: DES, Designation; PROG, Progression; WBC: White blood cell.

Table S2 Profiles of different AML subpopulations at the time of isolation and in culture.

DES	Initial subpopulations (At the time of isolation)				Cultured subpopulations in StemSpan (At the time of experiments)			
	$CD34^+$ $CD38^-$ (LSC)	$CD34^+$ $CD38^+$ (LPC)	$CD34^-$ $CD38^-$ (LB)	$CD34^-$ $CD38^+$ (pre-LB)	$CD34^+$ $CD38^-$ (LSC)	$CD34^+$ $CD38^+$ (LPC)	$CD34^-$ $CD38^-$ (LB)	$CD34^-$ $CD38^+$ (pre-LB)
Pr2R	7.1%	30.6%	60.0%	2.3%	37.1% ± 10.8%	7.8% ± 4.8%	52.6% ± 11.9%	2.6% ± 2.5%
Pr3R	68.6%	2.9%	22.1%	6.5%	94.4% ± 2.0%	1.4% ± 1.4%	0.8% ± 1.1%	3.4% ± 3.4%

Abbreviations: DES, Designation.

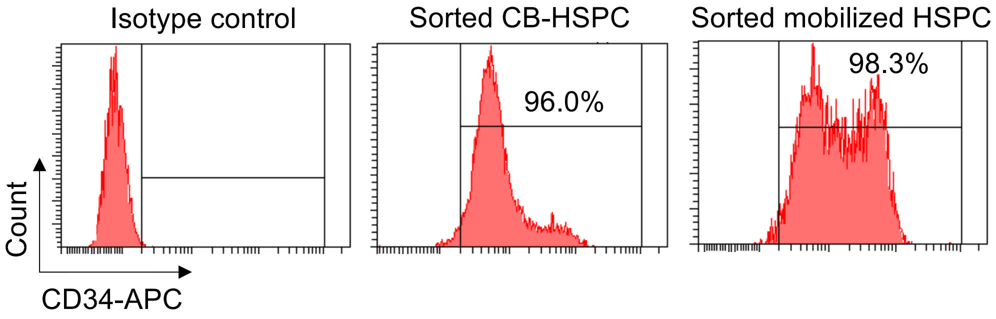

Fig. S1 Purity of CD34$^+$ HSPCs isolated from human cord blood and G-CSF-mobilized peripheral blood.

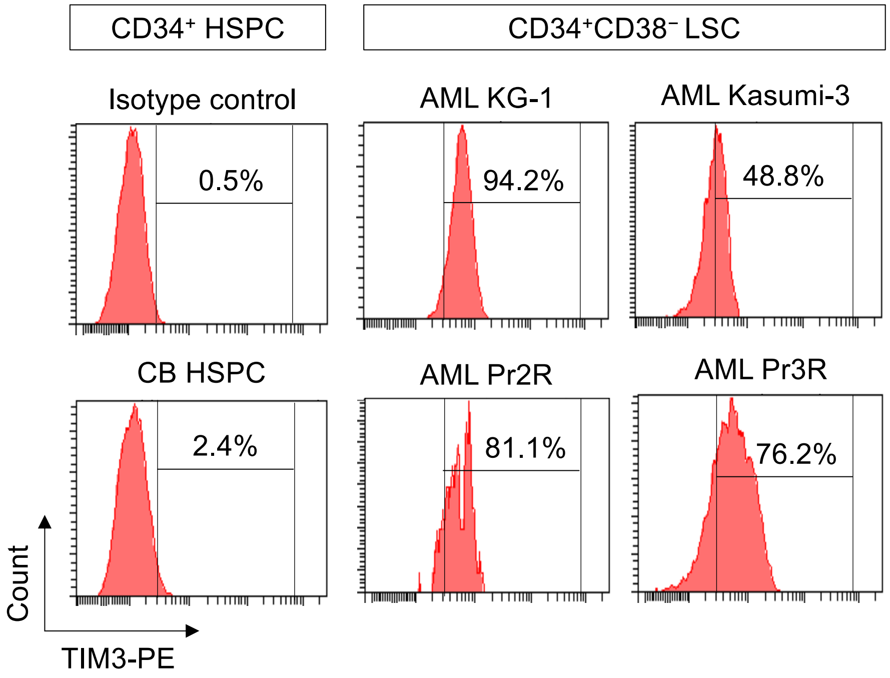

Fig. S2 TIM3 expression in LSCs and normal HSPCs. Flow cytometric analysis of TIM3 in CD34$^+$CD38$^-$ LSCs in human AML cell lines, KG-1 and Kasumi-3, and primary Pr2R and Pr3R cells in comparison to enriched CD34$^+$ HSPCs from cord blood (CB).

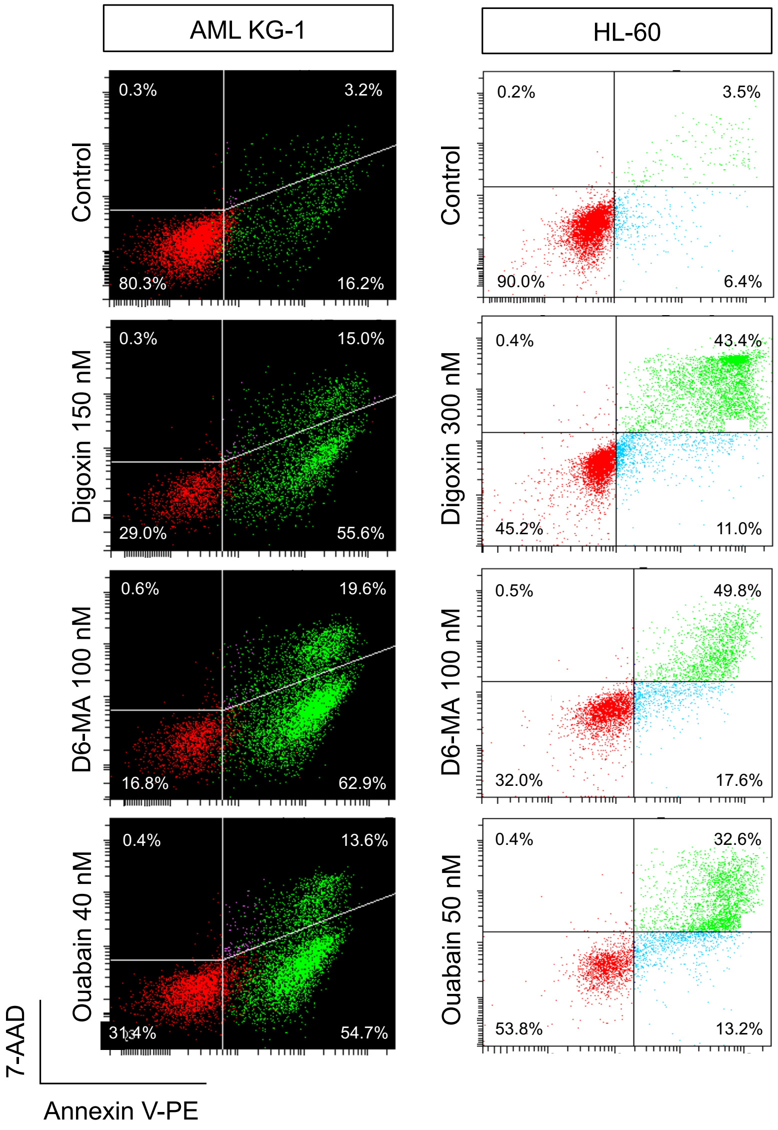

Fig. S3 Apoptosis is a major mode of cell death induced by cardiac glycosides in AML cells. AML KG-1 and HL-60 cells were treated with digoxin, D6-MA, and ouabain approximately at their LC50 (if available), and cell death was determined by flow cytometry using Annexin V/7-AAD assay. Representative dot plots from KG-1 (left) and HL-60 (right) cells are shown—Annexin V single-positive cells (low right quadrant) were defined as early apoptosis, Annexin V and 7-AAD double-positive cells (upper right quadrant) were defined as late apoptosis/necrosis, and 7-AAD single-positive cells (upper left quadrant) were defined as necrosis.

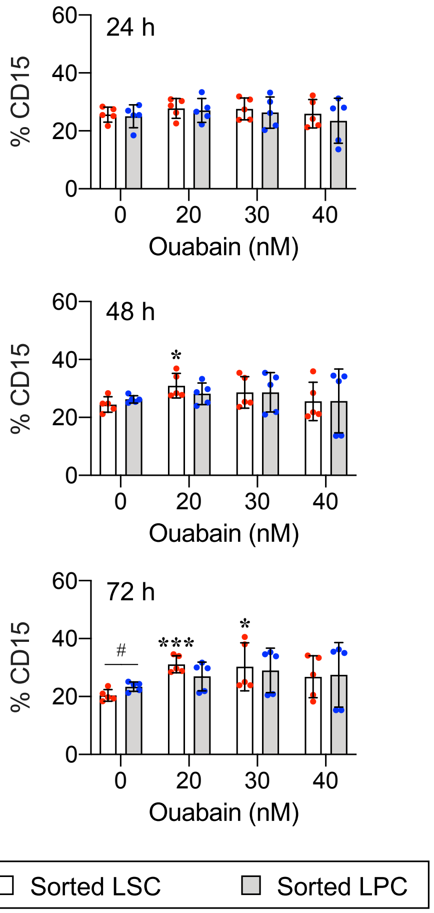

Fig. S4 Percentage of CD15^{+} blasts in live (Annexin V^{-}) cells upon ouabain treatment in study model 2. Enriched LSCs and LPCs from AML KG-1 cells were treated with ouabain (0–40 nM) for 24–72 h, and percentage of CD15^{+} blasts was determined by flow cytometry (see Fig. 4 for schematic illustration and gating strategy).

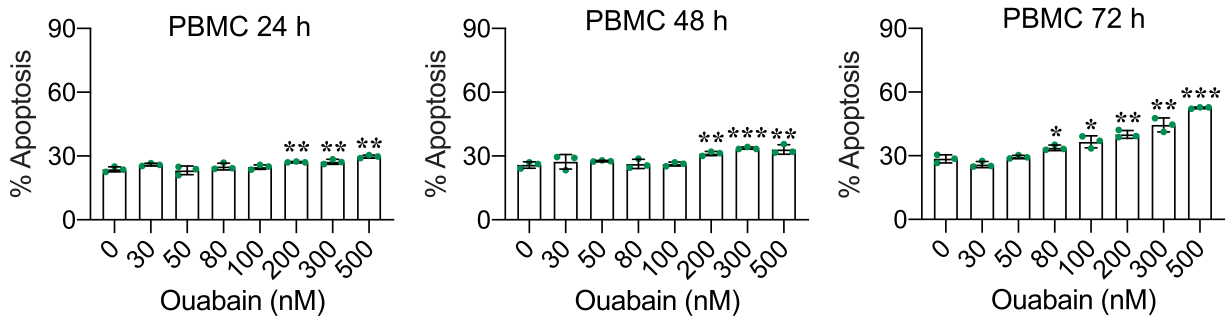

Fig. S5 Ouabain had minimal effect on normal PBMCs. Percentage of apoptosis as evaluated by Annexin V/7-AAD assay following ouabain treatment (0−500 nM) for 24−72 h in PBMCs obtained from healthy donors. Data are mean (n = 3). $^{*}P < 0.05$, $^{**}P < 0.01$, $^{***}P < 0.001$ versus nontreated control cells.

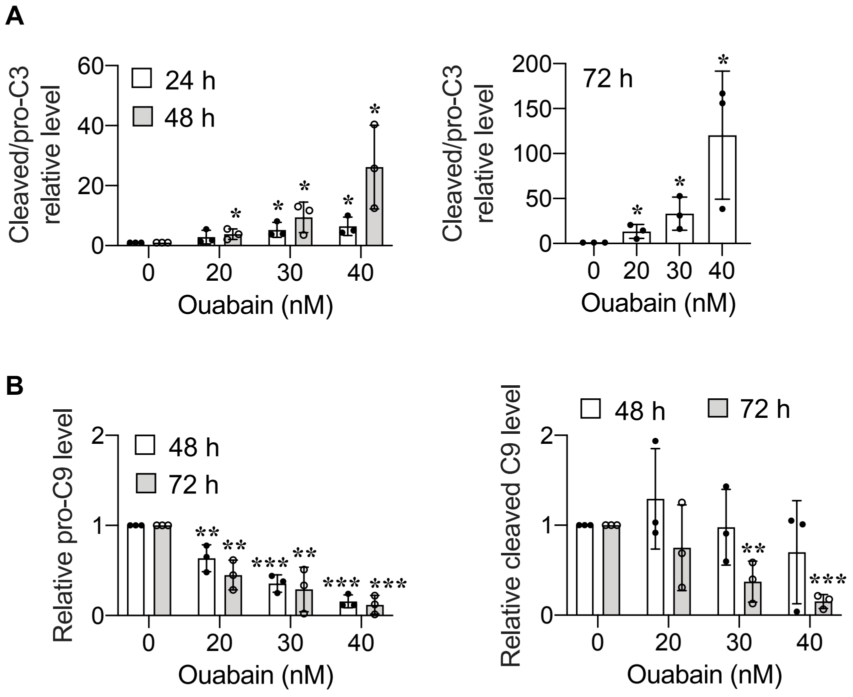

Fig. S6 Ouabain induces caspase activation in AML cells. **A** Densitometric analysis of caspase-3 (C3) activation presented as a ratio of cleaved to pro-C3 level, in correspond to immunoblots in Fig. 7C. Data are mean ± SD (n = 3). $^{*}P < 0.05$ versus nontreated control cells; two-sided Student's *t* test. **B** Densitometric analysis of pro-caspase-9 (C9) (left) and cleaved C9 (right) level, in correspond to immunoblots in Fig. 7C. Data are mean ± SD (n = 3). $^{**}P < 0.01$, $^{***}P < 0.001$ versus nontreated control cells; two-sided Student's *t* test.

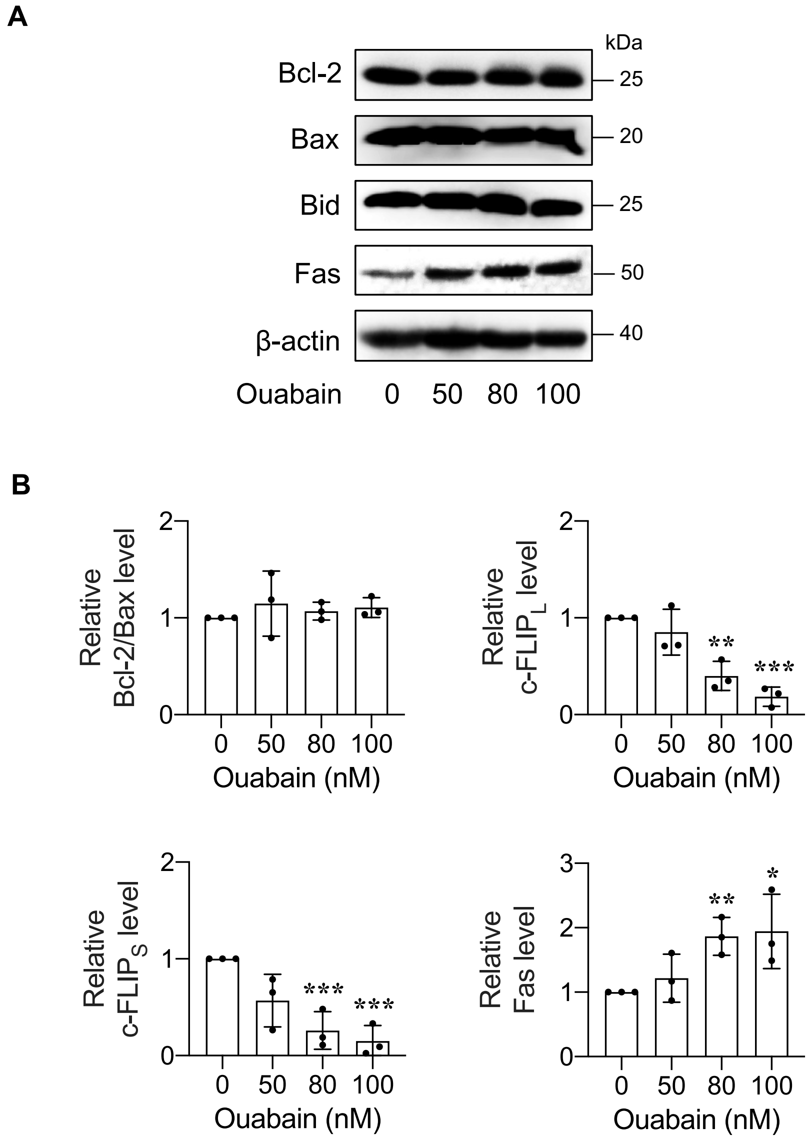

Fig. S7 Profiles of key apoptosis regulatory proteins in response to ouabain in AML HL-60 cells. **A** Western blot analysis of key apoptosis regulatory proteins, including Bcl-2, Bax, Bid and Fas, in addition to the profiles of Mcl-1, c-Myc, c-FLIP$_L$ and c-FLIP$_S$ in Fig. 9B. β-actin was used as a loading control. **B** Densitometric analysis of Bcl-2 and Bax presented as a ratio of Bcl-2 to Bax, c-FLIP$_L$ and c-FLIP$_S$ (in correspond to immunoblots in Fig. 9B), and Fas levels. Data are mean ± SD (n = 3). $^{**}P < 0.01$, $^{***}P < 0.001$ versus nontreated control cells; two-sided Student's *t* test.

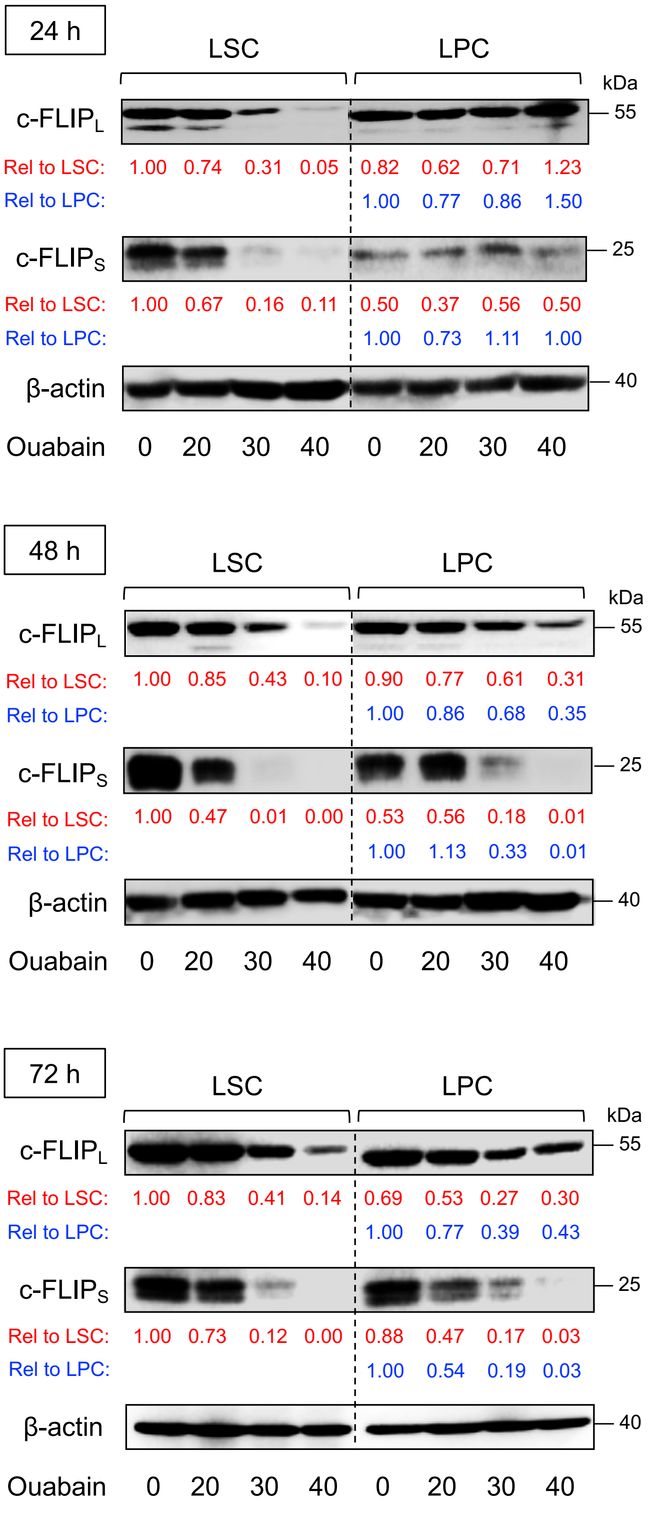

Fig. S8 Ouabain induces a rapid loss of c-FLIP$_L$ and c-FLIP$_S$ in LSCs. Kinetic analysis of c-FLIP$_L$ and c-FLIP$_S$ in enriched LSCs and LPCs from AML KG-1 cells following ouabain treatment (0–40

nM) for 24−72 h by Western blotting. β-actin was used as a loading control. Quantitative analysis of c-FLIP$_L$ and c-FLIP$_S$ levels is shown under the immunoblots. Dash line indicates juxtapose lanes taken from the same blots.

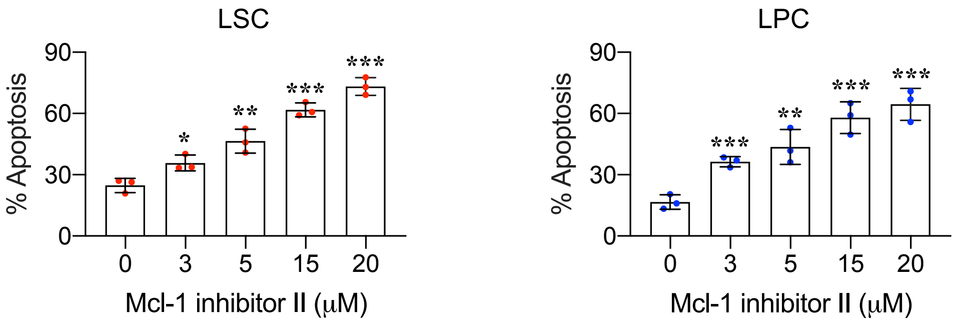

Fig. S9 Small molecule inhibition of Mcl-1 similarly caused apoptosis in LSCs and LPCs. Percentage of apoptosis as evaluated by Annexin V/7-AAD assay following Mcl-1 inhibitor II treatment (0−20 μM) in enriched LSCs (left) and LPCs (right) for 24 h. Data are mean (n = 3). $^{*}P < 0.05$, $^{**}P < 0.01$, $^{***}P < 0.001$ versus nontreated (vehicle; DMSO) control cells; two-sided Student's *t* test.
